# Parameters influencing the development of highly conductive and efficient biofilm during microbial electrosynthesis: the importance of applied potential and inorganic carbon source

**DOI:** 10.1038/s41522-020-00151-x

**Published:** 2020-10-14

**Authors:** Paniz Izadi, Jean-Marie Fontmorin, Alexiane Godain, Eileen H. Yu, Ian M. Head

**Affiliations:** 1 School of Engineering, Newcastle University, Newcastle upon Tyne, UK; 2 School of Natural and Environmental Sciences, Newcastle upon Tyne, UK; 3 Department of Chemical Engineering, Loughborough University, Loughborough, UK

**Keywords:** Biofilms, Water microbiology, Applied microbiology

## Abstract

Cathode-driven applications of bio-electrochemical systems (BESs) have the potential to transform CO_2_ into value-added chemicals using microorganisms. However, their commercialisation is limited as biocathodes in BESs are characterised by slow start-up and low efficiency. Understanding biosynthesis pathways, electron transfer mechanisms and the effect of operational variables on microbial electrosynthesis (MES) is of fundamental importance to advance these applications of a system that has the capacity to convert CO_2_ to organics and is potentially sustainable. In this work, we demonstrate that cathodic potential and inorganic carbon source are keys for the development of a dense and conductive biofilm that ensures high efficiency in the overall system. Applying the cathodic potential of −1.0 V vs. Ag/AgCl and providing only gaseous CO_2_ in our system, a dense biofilm dominated by *Acetobacterium* (*ca*. 50% of biofilm) was formed. The superior biofilm density was significantly correlated with a higher production yield of organic chemicals, particularly acetate. Together, a significant decrease in the H_2_ evolution overpotential (by 200 mV) and abundant *nifH* genes within the biofilm were observed. This can only be mechanistically explained if intracellular hydrogen production with direct electron uptake from the cathode via nitrogenase within bacterial cells is occurring in addition to the commonly observed extracellular H_2_ production. Indeed, the enzymatic activity within the biofilm accelerated the electron transfer. This was evidenced by an increase in the coulombic efficiency (*ca*. 69%) and a 10-fold decrease in the charge transfer resistance. This is the first report of such a significant decrease in the charge resistance via the development of a highly conductive biofilm during MES. The results highlight the fundamental importance of maintaining a highly active autotrophic *Acetobacterium* population through feeding CO_2_ in gaseous form, which its dominance in the biocathode leads to a higher efficiency of the system.

## Introduction

Over the last few decades, fossil fuels have been used as the main source of energy for human and industrial activities. The intense consumption of these resources has led to significant environmental effects including global warming and climate change^1^. CO_2_ emissions to the atmosphere from fossil fuel combustion is believed to be the main driver of these effects^2^. A range of approaches, including electrochemical methods, have been developed to reduce CO_2_ to valuable chemicals^3^. Over the past few decades, metal catalysts such as copper have been commonly used for electrochemical reduction of CO_2_ and have received lots of attention in this area^4–7^. Microbial electrosynthesis (MES) is a bio-electrochemical technique that has offered the sustainable conversion of CO_2_ to valuable organic chemicals at the cathode of bio-electrochemical systems (BES), using microorganisms as biocatalysts^8–10^. Despite the fact that electrochemical processes for CO_2_ reduction to date are much more mature and closer to industrial applications compared to MES, MES offers other advantages that are worth being considered such as lower over potential, and therefore lower energy consumption and more sustainable catalysts. In addition, the products in electrochemical CO_2_ reduction are typically limited to lower carbon products, particularly C1 compounds, formate and CO^11,12^. However, MES offers the opportunity to convert CO_2_ to more complex carbonaceous organic products. Currently, acetate synthesis through the Wood–Ljungdahl pathway has been the major product obtained in MES systems^13^. In addition, chain elongation from acetate for production of longer chain organics such as butyrate, caproate and their corresponding alcohols by MES from CO_2_ has been reported recently^14–22^. Despite the growing number of studies on MES, major bottlenecks exist in the development and scale up of the technology. One important limitation is the slow development of CO_2_-reducing biofilms on cathodes related to the low growth rate and yield of bacteria growing on the electrode surface and using the electrode as an electron donor and CO_2_ as a carbon source. This leads to the slow or sometimes no biofilm formation and thus slow start-up typically observed in MES processes^23,24^. In addition, the nature of the mechanisms (e.g. direct and indirect) involved in extracellular electron transfer from the electrode to microorganisms and the role of planktonic cells in MES have not been fully established. Several assumptions were made in previous studies, suggesting that MES processes would either be driven by planktonic bacterial community in the catholyte^25^, or only by the cathodic biofilm which would be responsible for CO_2_ conversion^19^. Through the direct interaction of microbial cells with the cathode, it was proposed that electrons are transferred directly from the cathode to the microbial community in the cathodic biofilm^8,26–28^. However, no specific pathway of direct electron transfer (DET) has been identified in MES systems. Nevertheless, indirect electron transfer (IET) has been observed in many studies, mainly with H_2_ as an intermediate electron carrier^28,29^. In previous studies on pure and mixed cultures of microorganisms, a potential of −0.6 V vs. Ag/AgCl was applied at the cathode to discard the possibility of abiotic H_2_ production. It was suggested that in the absence of H_2_ as an intermediate electron carrier, acetogens such as *Sporomusa ovata* and *Clostridium ljungdahlii* were able to take up electrons directly from the cathode^30–32^. However, even at −0.6 V it appears that electrons are still transferred between the cathode and CO_2_-reducing microorganisms such as methanogens through H_2_, produced enzymatically at the surface of the cathode, and consumed quickly by cathodic microorganisms^33^. The cathode potential is, therefore, a key parameter affecting the mechanism of electron transfer in MES systems^34^. As H_2_ production increases the electron transfer rate, rather than DET, production of medium and long chain organic compounds has been achieved in a number of studies by applying a negative potential (commonly more negative than −0.7 V vs. Ag/AgCl) at the cathode^19–21,35^. However, only a small number of these studies reported the formation of a cathodic biofilm in such conditions^19^. Dense and well-developed cathodic biofilms have been shown to significantly improve electron transfer rates and production rates in MES^36^, while lower production rates were observed in MES where planktonic cells dominated product formation^37^. In addition, MES driven by planktonic cells has the limitation that microbial communities can be washed out from the reactor by changing the operational mode to continuous feeding regime^25^. A significant increase (from 2.3 up to 11.8 fold) in acetate production rate was reported after the development of a dense *S. ovata* biofilm using a modified electrode^36,38–41^. However, number of studies have shown that only thin, monolayer biofilms can form on un-modified electrodes^42,43^. Due to the difficulties of enrichment of desirable bacteria from diverse microbial communities, it is essential to study the formation of well-developed biofilms from mixed inocula for MES to improve the applicability of this technique. In addition, in anodic biofilms typically dominated by *Geobacter*, cytochromes have been shown to play an important role in enhancing conductivity of the biofilm and subsequently the efficiency of the system^44,45^. However, electrochemical properties of acetogenic cathodic biofilms, typically dominated by *Acetobacterium* and their effect on MES is still not clear.

In addition to cathode potential, the form in which inorganic carbon is provided (e.g. HCO_3_^−^ or CO_2_) may also affect the progress of MES. In previous studies on MES, CO_2_, bicarbonate or both forms of inorganic carbon were provided in the catholyte^19,46–49^. However, its effect on biofilm formation and production through MES has not been investigated previously. Jourdin et al. reported the formation of mature biofilm on graphite felt fibres at an applied potential of −1.05 V vs. Ag/AgCl, however, because CO_2_ and bicarbonate were both used in the system, the effect of inorganic carbon source on biofilm formation could not be assessed^19^. Given the lack of detailed knowledge on the effect of inorganic carbon source on MES, a systematic investigation is required to determine to what extent the nature of the inorganic carbon source affects cathodic bacterial community composition and organic carbon production.

Due to the importance of energy and inorganic carbon sources in MES processes, the effect of these two parameters on biofilm development and the electrochemical properties and microbial composition of biofilms developed during MES, and consequently their effect on organic chemicals production were investigated in this study. Two different cathodic potentials (−0.8 V and −1.0 V) were selected and applied at the cathodes due to the presence and absence of abiotically produced H_2_ at these potentials. Gaseous CO_2_ or NaHCO_3_ were used to test the effect of inorganic carbon source on MES processes. We demonstrated that supplying gaseous CO_2_ and applying a potential of −1.0 V at the cathode led to the development of highly conductive and efficient well-formed biofilms which remained highly active during 104 days of the experiment even under acidic pH due to products accumulation. Electrochemical and morphological properties of the mature biofilm formed during MES processes as well as the microbial composition of the biofilm were studied. The potential mechanisms of electron transfer between the cathode and microbial cells and organic compound synthesis were also investigated by shotgun metagenome sequencing.

## Results and discussion

### Effect of cathode potential on organic compounds production by MES

#### Current consumption and acetate production from CO_2_ at cathodic potentials of −1.0 V and −0.8 V (BES 1 and BES 3)

**Fig. 1:**
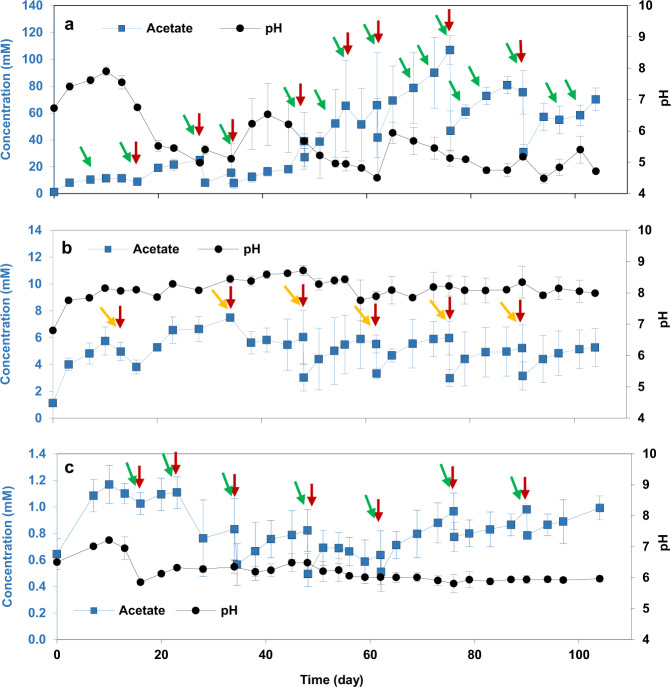
The effect of cathodic applied potential and inorganic carbon source on pH and acetate production. Catholytes pH and concentration of acetate in **a** BES 1 (−1.0 V/CO_2_), **b** BES 2 (−1.0 V/NaHCO_3_), and **c** BES 3 (−0.8 V/CO_2_) during 104 days of the experiment through MES. The error bars represent the standard deviation calculated from duplicate samples in two reactors. The red, green and orange arrows indicate when fresh medium, CO_2_ and bicarbonate were provided, respectively. CO_2_ was purged in the medium when the inorganic carbon concentration was negligible.

Before starting BES, an abiotic control experiment was carried out for 13 days to evaluate the possible production of H_2_ at −1.0 V (BES 1) and −0.8 V (BES 3). CO_2_ was the carbon source in both conditions. The cathodic current during abiotic control was around −0.01 and −0.03 mA cm^−2^ at the applied potential of −0.8 and 1.0 V, respectively (Supplementary Fig. 1). Subsequently, no H_2_ was detected in the headspace of the reactors poised at −0.8 V over a 13 day period, however, 1.3 ± 0.3 ml H_2_ was detected in the headspace of reactors at −1.0 V. No other gas products were detected in the headspaces of the reactors. The possibility of abiotic H_2_ evolution on a graphite felt cathode poised at −1.0 V was confirmed by CV of a plain graphite felt electrode (see CV results). In addition, no organic products were detected in the catholyte, showing that abiotic reduction of CO_2_ does not occur at these potentials. After starting the biotic experiment, to test the effect of cathode potential on current consumption and acetate production through MES, two reactors, BES 1 and BES 3, were operated at −1.0 V and −0.8 V, respectively for 104 days. Effluent from an operating BES biocathode, producing acetate as the sole product (7.5 ± 1.1 mM), was used as inoculum. In each freshly inoculated BES, cathodic current started within 1 day of inoculation, most significantly in BES 1 (−1.0 V/CO_2_). A maximum current density obtained in BES with different cathode potentials ranged from –0.007 to –1.8 mA cm^−2^ (Supplementary Fig. 2). Cathodic current in BESs were higher than abiotic control, more significantly in BES 1 (−1.0 V/CO_2_). Cathodic current in BES 1 (−1.0 V/CO_2_) began at −0.9 ± 0.2 mA cm^−2^ and stayed relatively stable over the first 20 days. From day 21, cathodic current increased and reached the maximum of −1.8 ± 0.3 mA cm^−2^ on day 60. Conversely, cathodic current was negligible in BES 3 (−0.8 V/CO_2_; maximum of −0.03 ± 0.0 mA cm^−2^) throughout the experiment. Acetate was the major product produced in all BESs (Fig. 1). Acetate concentration in BES 3 (−0.8 V/CO_2_; maximum 1.2 ± 0.1 mM) was negligible compared to BES 1 (−1.0 V/CO_2_; Fig. 1). In BES 1 (−1.0 V/CO_2_), acetate concentration increased gradually from the beginning until day 40, reaching maximum concentration and production rate of 19.1 ± 2.6 mM and 2.6 ± 0.3 mM day^−1^, respectively (Fig. 1 and Supplementary Fig. 3). However, after day 40, the acetate concentration increased significantly, reaching a maximum production rate of 11.0 ± 1.6 mM day^−1^ on day 54 and a concentration of 106.9 ± 10.5 mM on day 74. Similarly, coulombic efficiency of the system through acetate production over the first 20 days of experiment was 51.5 ± 19.4 %. The rest of the electrons from the cathodic electrode was possibly consumed for biomass production. Although no H_2_ was detected in the headspaces, it was also possible that electrons were consumed for H_2_ production which were not used by microbial communities for MES over the first 20 days and consumed by other hydrogenotrophic microorganisms. Coulombic efficiency increased to *ca*. 69% between days 40 and 63 and remained relatively stable until the end of the experiment, which is comparable with previous reports^50^.

#### Production of longer chain organic compounds than acetate from CO_2_ at a cathode potential of −1.0 V and −0.8 V (BES 1 and BES 3)

**Fig. 2:**
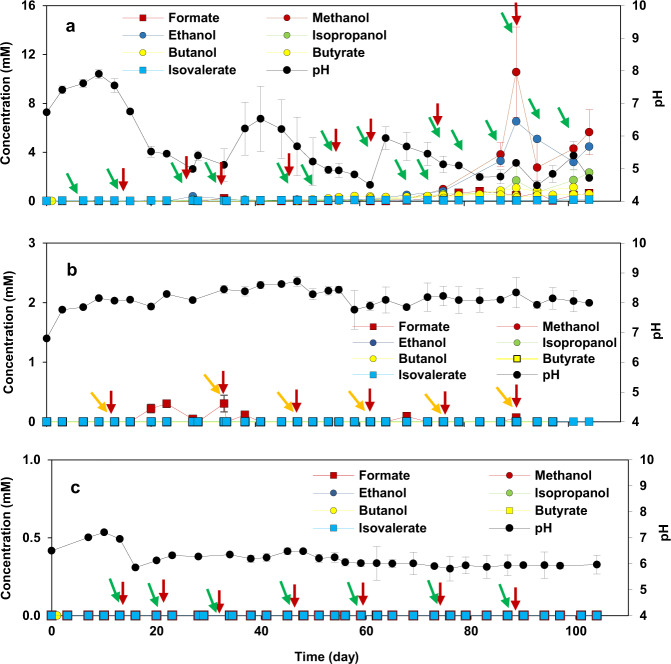
The effect of cathodic applied potential and inorganic carbon source on production of longer chain compounds than acetate. Concentration of longer chain carboxylic acids than acetate and alcohols in **a** BES 1 (−1.0 V/CO_2_), **b** BES 2 (−1.0 V/NaHCO_3_), and **c** BES 3 (−0.8 V/CO_2_) during 104 days of the experiment through MES. The error bars represent the standard deviation calculated from duplicate samples in two reactors. The red, green and orange arrows indicate when fresh medium, CO_2_ and bicarbonate were provided, respectively. CO_2_ was purged in the medium when the inorganic carbon concentration was negligible.

Decrease in the catholyte’s pH of BES 1 (−1.0 V/CO_2_) after day 58 caused by accumulated acetate (Fig. 1) led to production of longer chain organic compounds (Fig. 2a). The production of more diverse products including butyrate and isovalerate after day 58 was associated with a decrease in production rate and concentration of acetate, suggesting chain elongation may have been driven by acetate incorporation. The Wood–Ljungdahl pathway plays a key role in the production of acetate in MES by fixing CO_2_^51^. Production of organic acids from CO_2_ by acetogens through this pathway occurs by synthesizing acetyl-CoA from CO_2_ reduction^52^. The production of butyrate can occur either via linear extension of the acetyl-CoA to butyryl-CoA, or via reverse β-oxidation known as microbial chain elongation^25^. Odd-chain carboxylates production was reported through propionyl-CoA pathway^53^. In case of incorporation of propionyl-CoA in place of acetyl-CoA in the initial steps of carboxylates synthesis, propionate can be elongated to C5 products^54^. Although no propionate was detected in BES 1 (−1.0 V/CO_2_), it could be elongated to isovalerate using ethanol^55^ or as reported recently using methanol under acidic condition (pH of 5.5 to 5.8) by Clostridium^56^. Propionate production could be related to Prevotella^57^, a propionate-producing bacteria, found in the microbial communities of BES 1 (−1.0 V/CO_2_) (see ‘Cathodic biofilm and catholyte microbial communities’ section).

Alongside the production of carboxylic acids, production of alcohols such as methanol, ethanol, isopropanol and butanol was observed in BES 1 (−1.0 V/CO_2_) after around 60 days. Ethanol and butanol were produced after day 60, with maximum concentrations of 6.5 ± 1.8 mM at day 90 and 1.2 ± 0.0 mM at day 104, respectively. Detection of alcohols in the catholyte was consistent with the drop in pH measured due to the accumulation of acidic products during the same period. The pH drop and alcohol production indicates the switch in metabolism from acetogenesis to solventogenesis, leading to the production of solvents, such as alcohols. During solventogenesis also called as ‘’acid crash”, acetogens such as some *Clostridium* spp. re-assimilate the products and reduce them to their corresponding alcohols such as ethanol or butanol^20,58^. Ethanol and butanol are typically the main products of solventogenesis^59^. Although alcohols were detected in BES 1 (−1.0 V/CO_2_), their concentrations were almost negligible compared to the previous study^60^, indicating that solventogenesis was not dominating the microbial metabolism. Intermittent detection of methanol or ethanol during the experiment likely resulted from their consumption required for production of longer chain organic compounds such as butyrate and isovalerate detected in BES 1 (−1.0 V/CO_2_)^54,61,62^. These observations are in agreement with a previous study on acetate production via MES which reported that significant ethanol and butyrate production began when the accumulated acetate concentration reached more than 1500 ppm and the pH of the catholyte was slightly acidic^63^. In addition, isopropanol (maximum of 2.3 ± 0.1 mM) was detected in the present study when small concentrations of acetone were occasionally detected. This was in agreement with Arends et al.^25^ who demonstrated that acetone, produced in the acetone-butanol-ethanol (ABE) fermentation, was important for isopropanol production in an isopropanol-butanol-ethanol (IBE) fermentation^25^. Despite the significant production of longer chain products in BES 1 (−1.0 V/CO_2_), no long chain organic compounds were detected in BES 3 (−0.8 V/CO_2_) where acetate production was negligible (Fig. 2). Similarly, no production of organic compounds was observed in reactors under open circuit potential (OCP) conditions, showing the key role of a polarized cathode in providing energy for MES. The H_2_ produced extracellularly was an electron mediator which was consumed by microbial communities for CO_2_ reduction and organics production through MES. Therefore, the lack of H_2_ production in BES 3 (−0.8 V/CO_2_) and OCP control reactors resulted in inferior organic compound production through MES.

### Effect of inorganic carbon source on organic compounds production by MES

#### Current consumption and organic compounds production from gaseous CO_2_ and bicarbonate at cathodic potential of −1.0 V (BES 1 and BES 2)

In order to compare the impact of different types of inorganic carbon source on production through MES, the performance of BES 1 (−1.0 V/CO_2_) and BES 2 (−1.0 V/NaHCO_3_) was compared. Compared to BES 1 (−1.0 V/CO_2_), cathodic current was lower in BES 2 (−1.0 V/NaHCO_3_), with a maximum of −0.2 ± 0.0 mA cm^−2^ (Supplementary Fig. 2). In addition, acetate concentration in BES 2 (−1.0 V/NaHCO_3_) was much lower than that in BES 1 (−1.0 V/CO_2_) over the 104 day duration of the experiment (Fig. 1b), with maximum acetate concentration of 7.4 ± 0.1 mM on day 34 and maximum acetate production rate of 0.26 ± 0.03 mM day^−1^ on day 103 (Supplementary Fig. 4). The superior performance of BES 1 (−1.0 V/CO_2_) compared to BES 2 (−1.0 V/NaHCO_3_), could be attributed to the pH of the catholyte. Although pH of the catholyte was adjusted to 6.9 ± 0.1 at every medium change, the pH rapidly increased to between 7.5 and 8.5 in BES 2 (−1.0 V/NaHCO_3_), after less than one day following the medium change. By contrast there was a significant drop in pH in BES 1 (−1.0 V/CO_2_) at day 20 to around 5.0. The pH remained relatively stable between 7.5 and 8.5 in BES 2 (−1.0 V/NaHCO_3_) probably due to the buffering power of bicarbonate (pH **=** 8.3). Unlike in BES 1 (−1.0 V/CO_2_) longer chain organic acids and alcohols were not observed in BES 2 (−1.0 V/NaHCO_3_) (Fig. 2b). This was attributed to the low concentration of acetate and high pH in the catholyte of BES 2.

#### Metabolism of CO_2_ reducing bacteria

The optimal pH for acetogens is slightly acidic (*ca*. 6.0)^64,65^. This explains the similar acetate production in BES 1 (−1.0 V/CO_2_) and BES 2 (−1.0 V/NaHCO_3_) over the first 20 days of the experiment, when the pH was *ca*. 7.0 in both treatments. The near neutral catholyte pH in BES 2 (−1.0 V/NaHCO_3_) throughout the experiment may have prevented strong enrichment of acetogenic bacteria and consequently led to lower production of acetate and longer chain organic compounds. The detection of H_2_ (*ca*. 1.4 ml in the headspace) in the headspaces of the BES 2 reactors (−1.0 V/NaHCO_3_) demonstrated that H_2_ was not consumed by the bacterial community for MES. By contrast H_2_ was never detected in the headspace of BES 1 (−1.0 V/CO_2_) during the 104-day experiment.

Total inorganic carbon (TIC) was measured periodically throughout the experiment to record the inorganic carbon consumption. TIC decreased in every batch cycle (3–5 days) in BES 1 (−1.0 V/CO_2_) and reached the negligible concertation. However, the decrease in TIC in BES 2 (−1.0 V/NaHCO_3_) was observed in more than 10 days. In addition in BES 2 (−1.0 V/NaHCO_3_), not all the bicarbonate was consumed at the end of the batch cycles. While it seemed all the CO_2_ was converted to acetate and other products in BES 1 (−1.0 V/CO_2_), 67.9 ± 15.3% was the average of carbon conversion efficiency during 104 days of experiment in BES 2 (−1.0 V/ NaHCO_3_). The calculation details of carbon conversion efficiency is provided in the supplementary information. This suggested that the acetogens in the BES could not utilize CO_2_ and HCO_3_^−^ equally well. It was shown previously that acetogens can consume bicarbonate and/or CO_2_ through MES successfully^19,66,67^. However, bicarbonate consumption was not compared with CO_2_ consumption through MES systematically. Therefore, it is still not clear in what extend the form of inorganic carbon source could affect the MES processes. In our study, higher rate of CO_2_ consumption was observed compared to bicarbonate consumption. Therefore, it is highly likely that the acetogens are in favour of utilizing CO_2_ than bicarbonate. *Clostridium kluyveri* is known to need CO_2_ for protein synthesis^68^. It was concluded that CO_2_ as an inorganic carbon source may lead to higher rate of protein synthesis than HCO_3_^−^^69^. At pH values higher than 7.0, almost all inorganic carbon is in the form of bicarbonate, and low bicarbonate consumption and low production of organic products were observed^69^. At pH values around 6.0, *ca*. 50% of dissolved inorganic carbon is in the form of CO_2_^69^. This is consistent with our observation that acetate production was much greater in BES 1 (pH 6.0) than in BES 2 (pH 8.0).

#### Competition between acetogens and methanogens

Despite the advantages associated with utilisation of a mixed microbial community for MES applications (e.g. increased diversity of microorganisms in biofilms and their synergistic effects), it potentially has detrimental effects caused by competition for hydrogen consumption between acetogens and hydrogenotrophic methanogens and acetate consumption by acetocalstic methanogens^70,71^. Several approaches to inhibit the activity of methanogens have been suggested. These include treatment with chemical inhibitors of methanogens (e.g. 2-bromoethanesulfonic acid), temperature control, heat treatment of inocula to kill methanogens and select for spore-forming acetogens and operation at low pH^72^. In this study, although the initial inoculum was pre-heated, 2–4% methane was detected in the headspaces of BES 1 (−1.0 V/CO_2_) and 5% methane was detected in the headspace of BES 2 (−1.0 V/NaHCO_3_) over the first 30 days of the experiment. Therefore, 2-bromoethanesulfonate (5 mM) was added to the medium on day 30 to inhibit methanogens. No methane was detected in the headspace of the BES from day 30 until the end of the experiment. In addition, after the significant drop of pH from 6.5 to around 5.0 in BES 1 (−1.0 V/CO_2_) after 48 days of operation, 2-bromoethanesulfonate was no longer added to the medium and methane was not detected subsequently in BES 1. Therefore, low pH, and the associated higher CO_2_ and lower HCO_3_^−^ as well as enhancement of hydrogen production (higher proton reduction at lower pH), could be key parameters contributing to superior production of organic compounds in BES 1 (−1.0 V/CO_2_) compared to BES 2 (−1.0 V/NaHCO_3_).

### Morphological properties of cathodic biofilms catalysing MES

#### SEM observations

**Fig. 3:**
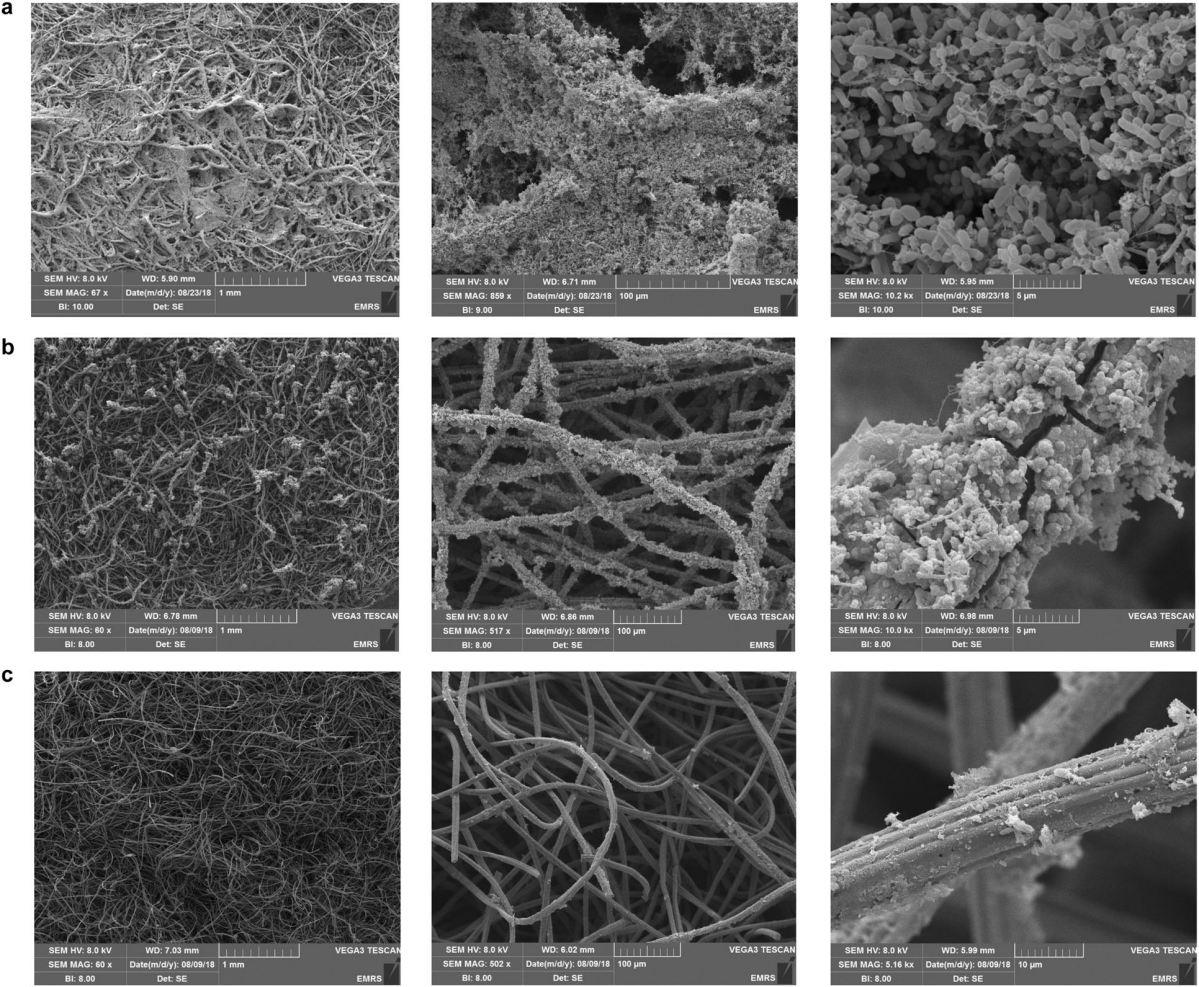
The effect of cathodic applied potential and inorganic carbon source on biofilm formation at the cathode. SEM images of cathodes following 104 days of operation in **a** BES 1 (−1.0 V/CO_2_), **b** BES 2 (−1.0 V/NaHCO_3_) and **c** BES 3 (−0.8 V/CO_2_). From left to right the scale bars represent 1mm, 100 microns and 5 or 10 microns, respectively.

Biofilms formed on BES cathodes with different cathode potentials and different inorganic carbon sources were examined by scanning electron microscopy (SEM) at the end of the experiment (day 104) after terminating the reactors. Complete coverage of the graphite felt by biofilm was observed at the electrodes of BES 1 (−1.0 V/CO_2_) (Fig. 3a). In places, the biofilm spanned individual graphite fibres. Biofilms of this nature have been referred to as “filamentous biofilm”^19^. Jourdin et al.^19^ observed similar electrode coverage by biofilm from a BES operated at a cathode potential of −1.05 V^19^. According to the authors, the long-term, continuous operation, and microbial inoculum source were important for biofilm development. On electrodes from BES 2 (−1.0 V/NaHCO_3_) (Fig. 3b), a biofilm was observed on individual graphite felt fibres but at a lower density than that in BES 1(−1.0 V/CO_2_). However, cathodes from BES 3 (−0.8 V/CO_2_) (Fig. 3c) displayed almost no biofilm on the graphite fibres, with few isolated bacterial cells observed. This was similar to the coverage of cathodes from BES operated under OCP conditions (Supplementary Fig. 6) and not dissimilar to plain electrodes (Supplementary Fig. 7).

#### Confocal microscopy observations

**Fig. 4:**
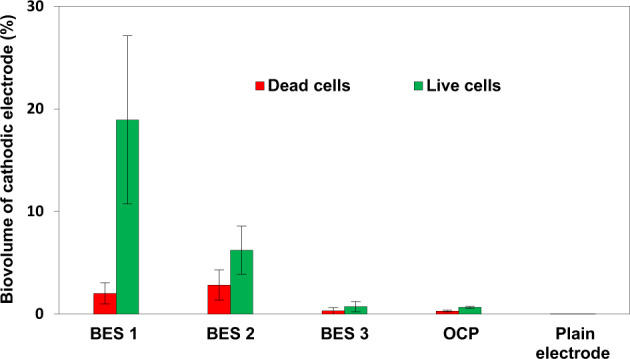
Relative abundance of live and dead cells in cathodic biofilms from BES 1 (−1.0 V/CO_2_), BES 2 (−1.0 V/NaHCO_3_), BES 3 (−0.8 V/CO_2_), OCP control reactors and plain graphite felt electrode. The error bars represent the standard deviation calculated from 3 fields of view from duplicate samples from two reactors (*n* = 12).

To obtain more detailed and quantitative information about cathodic biofilm formation and to determine the relationship between cathodic biofilm formation on the surface of graphite felt and production of organic compounds by MES, confocal microscopy was performed. Samples were taken from BES electrodes operated at different cathode potentials and with different inorganic carbon sources at the end of the experiment (day 104), which had been stained with a Live/Dead fluorescent stain (Fig. 4). The highest coverage of graphite fibres with biofilm occurred on the electrodes from BES1 (−1.0 V/CO_2_). The cathode from BES 1 (−1.0 V/CO_2_) had a total biofilm coverage of 19.1 ± 8.3 %. This had the highest proportion of live cells (*ca*. 90% of the biofilm was comprised of live cells) (Fig. 4). Total biofilm coverage of cathodes from BES 2 (−1.0 V/NaHCO_3_) was lower (8.5 ± 3.9%) in which *ca*. 66% of biofilm was comprised of live cells. Thus biofilm growth was greater when inorganic carbon was supplied in form of CO_2_ in the solution. Comparing electrodes poised at −1.0 V (BES 1) and −0.8 V (BES 3) both fed by CO_2_, it can be seen that the percentage of biofilm coverage on the electrode poised at −0.8 V was much lower (1.1 ± 0.7% biofilm coverage), and similar to the biofilm coverage obtained under OCP condition (Fig. 4).

#### Correlation between biofilm formation and BES performance

Bio-imaging results highlighted two important findings. Firstly, it demonstrated that BES with greater production of organic compounds had cathodic biofilms with a higher density, confirming the previous observations^38,40,73^. However, modified electrodes were used in these studies to enhance the biofilm formation. In our study, the densest biofilm was formed on the cathodes of BES 1 (−1.0 V/CO_2_) which had the highest percentage of live cells and, the highest concentration and most diverse range of organic products. In addition, regardless of inorganic carbon source, complete coverage of the graphite fibre in BES 1 (−1.0 V/CO_2_) and BES 2 (−1.0 V/NaHCO_3_) showed that biofilm formation was most pronounced at a cathode potential of −1.0 V. Although the thickness of the biofilm was higher in BES 1 (−1.0 V/CO_2_), biofilm was distributed similarly in BES 1 (−1.0 V/CO_2_) and BES 2 (−1.0 V/NaHCO_3_) (Fig. 3). The lower density of biofilm formed on the cathodes of BES 2 (−1.0 V/NaHCO_3_) seemed to be related to the use of bicarbonate as an inorganic carbon source as discussed before.

No biofilm was observed on cathodes with the applied potential of −0.8 V or at OCP (Figs. 3 and 4). The lack of abiotic H_2_ production at −0.8 V is likely to have resulted in minimal bacterial growth emphasising the effect of applied potential on cathodic biofilm formation and the important role of H_2_ as a mediator in electron transfer.

Absolute numbers of cells (live and dead) were also determined by flow cytometry. Flow cytometry analysis of BES 1 (−1.0 V/CO_2_), BES 2 (−1.0 V/NaHCO_3_) and BES 3 (−0.8 V/CO_2_) showed the highest number of cells was in the biofilm and catholyte of BES 1 (Supplementary Fig. 9).

### Electrochemical properties of biofilms formed during MES

#### H_2_ production in BES

**Fig. 5:**
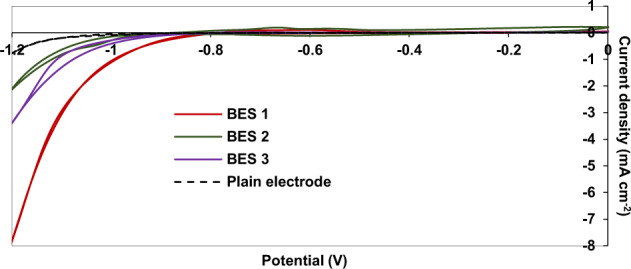
Cyclic voltammograms recorded at scan rate of 2 mV s^−1^. CVs are shown for a plain graphite felt electrode and cathodes from BES 1 (−1.0 V/CO_2_), BES 2 (−1.0 V/NaHCO_3_), and BES 3 (−0.8 V/CO_2_) after 104 days of operation.

Given the evidence from microscopy and formation of mature biofilm at the surface of the cathode of BES 1 (−1.0 V/CO_2_) in particular and the differences in organic compound production between treatments, it is important to determine the role of the biofilm in transfer of electrons and reduction of CO_2_. Cyclic voltammetry (CV) was conducted after 104 days of operation of the BES and compared with the CV from the plain electrode at the beginning of experiment. CV was performed after changing the medium and adjusting the pH to 6.5. A significant shift in the onset potential of the hydrogen evolution reaction (HER) was observed in all the BES compared to the plain graphite felt electrode (Fig. 5). The current density increased at potentials more negative than −1.0 V for electrodes from all BES (Fig. 5). By contrast, the onset HER potential was around −1.0 V on the plain electrode (Fig. 5). The onset potential was around −0.9 V for BES 2 (−1.0 V/NaHCO_3_) and BES 3 (−0.8 V/CO_2_) and was as high as −0.8 V in BES 1 (−1.0 V/CO_2_) (Fig. 5). A small oxidation peak appeared in voltammograms of BES 1 (−1.0 V/CO_2_) and BES 2 (−1.0 V/NaHCO_3_) around −0.6 V which was more likely associated with H_2_ production, as the peak disappeared after limiting the reductive potential of the forward scan to −0.8 V instead of −1.2 V. Given this observation one might have expected H_2_ production in BES 1 (−1.0 V/CO_2_). However, no H_2_ was detected in the headspaces of BES 1 (−1.0 V/CO_2_). This was more likely due to its consumption as an energy source by the hydrogenotrophic microbial communities. While still observed, the shift in onset potential of the HER was much less in BES 2 (−1.0 V/NaHCO_3_) and BES 3 (−0.8 V/CO_2_), which correlates with low H_2_ evolution and low levels of organic compound production in these BES.

In addition, the correlation between the thickness of the biofilm in BES 1 (−1.0 V/CO_2_; observed by microscopy) and the significant shift in the onset potential for the HER strongly suggests that the biofilm is responsible for the higher onset potential for the HER and that biofilm growth was promoted by the enhanced production of H_2_. This observation also confirms the key role of H_2_ as a mediator between the electrode and the microbial community and this is likely the dominant mechanism of electron transport driving MES in our study. At sufficiently negative cathode potentials, the abiotic formation of H_2_ or formate at the surface of graphite electrodes is thermodynamically favourable, but it is kinetically slow^8^. However, the involvement of extracellular enzymes or coenzymes from acetogens or methanogens such as hydrogenases, formate dehydrogenase or cytochromes are known to catalyse the formation of small compounds such as H_2_ or formate by interacting with the surface of a polarised cathode^74^. Duetzmann et al. investigated the electron uptake from the electrode by a pure culture of a methanogen (*M. maripaludis)* at a poised potential of −0.8 V^33^. They noted that enzymes, such as hydrogenases or formate dehydrogenases were secreted into the medium and catalysed the evolution of H_2_ or formate as a mediator, refuting direct intercellular electron transfer (DIET) as the mechanism through which cathodic CO_2_ reduction occurs. In this system the hydrogen and formate are rapidly consumed by microorganisms, mimicking DIET^33^. Although in our study a contribution of DIET between the cathode and bacterial community could not be ruled out completely, it is evident that the extensive biofilm formed on cathodes poised at −1.0 V was related to production of H_2_, confirming H_2_-mediated electron transfer as a dominant mechanism driving MES in this system. Moreover, the small amount of H_2_ production on the plain electrode poised at −1.0 V, observed in abiotic control experiments could initiate cathode colonization and start-up MES in BES 1 and BES 2.

#### Significant conductivity of the dense cathodic biofilm in BES 1 (−1.0 V/CO_2_)

**Fig. 6:**
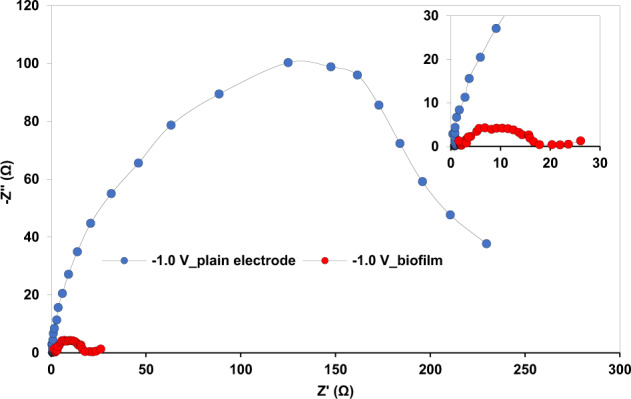
Nyquist plots at a potential of −1.0 V for a plain electrode and the cathode of BES 1 (−1.0 V/CO_2_) following 104 days of operation. The inset shows a close up of the main figure.

Although cathodic biofilms are known to catalyse CO_2_ reduction, one of the concerns regarding biofilm growth is diffusion limitation or charge transfer resistance caused by a dense biofilm^75^. For anodic biofilms, it has been reported that a dense biofilm can lead to an increase in electron transfer resistance between the microbial community and the anode^76^. To assess this in our cathodic biofilms, we used EIS to compare electron transfer resistance between cathodes with the densest biofilms (BES 1, −1.0 V/CO_2_) and plain graphite electrodes. The mechanism steps of electron transfer in biocathode were reported to be likely similar to bioanode including electron transfer from the cathode through a layer, and following that, layers of microorganisms^77^. A model reported by a Ter Heijne et al.^77^ from EIS response for oxygen reduction reaction in aerobic biocathode included charge transfer resistance, double layer capacitance, diffusion and ohmic resistance. Charge transfer resistance represents the electron transfer kinetics driven by the overpotential. This was more than 200 Ω at the plain electrode poised at −1.0 V in our study (Fig. 6). The variation in Nyquist plots after biofilm development at the cathode indicated the effect of biochemical reactions in enhancing the electron transfer between the cathode and microorganisms (and CO_2_) (Fig. 6). It was in fact observed that charge transfer resistance decreased by approximately 10 times after the development of the dense cathodic biofilm (Fig. 6). To the best of our knowledge, this is the first time that a significant decrease in charge transfer resistance has been reported for a CO_2_-reducing biofilm. This suggests that the dense biofilm formed on BES 1 (−1.0 V/CO_2_) cathodes was highly conductive facilitating electron transfer between the cathode and CO_2_-reducing microorganisms. Given the evidence from CV for biologically enhanced H_2_ production (see CV results), improved charge transfer through the biofilm (Fig. 6) is related to biotic H_2_ evolution.

### Cathodic biofilm and catholyte microbial communities

**Fig. 7:**
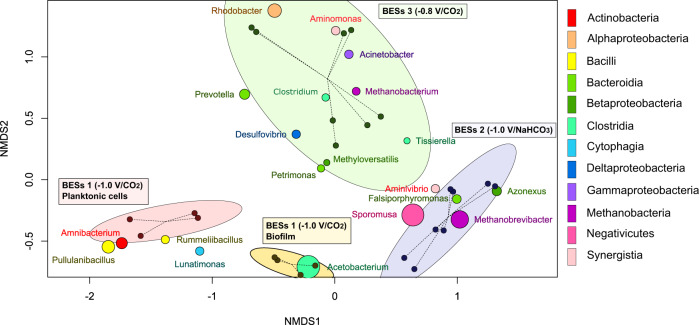
Non-metric multidimensional scaling (NMDS) of a Bray–Curtis distance matrix with 4 clusters of BES 1 (−1.0 V/CO_2_) planktonic cells, BES 1 (−1.0 V/CO_2_) biofilm, BES 2 (−1.0 V/NaHCO_3_) and BES 3 (−0.8 V/CO_2_) and 20 genera with the highest abundance in communities of all the samples. The diameter of each cluster represents the 90% confidence interval from the centre of the samples (stress = 0.08795, *R*^2^ = 0.96).

**Fig. 8:**
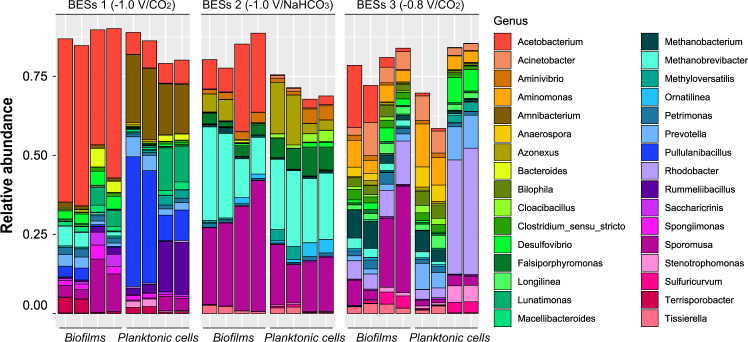
Relative abundance of bacterial community at the genus level obtained from Illumina sequencing of 16S rRNA genes from electrodes and planktonic cells from the catholyte in BES 1 (−1.0 V/CO_2_), BES 2 (−1.0 V/NaHCO_3_) and BES 3 (−0.8 V/CO_2_). Each group of four stacked bars shows microbial community profiles from duplicate samples from each of two replicate BES.

After 104 days of operation, the microbial communities in cathodic biofilms and the catholyte were characterized by 16S rRNA gene sequencing. Non-metric multidimensional scaling (NMDS) analysis of microbial community profiles revealed four different clusters (MANOVA test: *p*-value = 1.0 × 10^−3^, *R*^2^ = 0.65) corresponding to BES 1 (−1.0 V/CO_2_) planktonic cells, BES 1 (−1.0 V/CO_2_) biofilm, BES 2 (−1.0 V/NaHCO_3_) and BES 3 (−0.8 V/CO_2_). The cathode biofilm and catholyte communities from BES 2 and BES 3 were recovered together in single relatively large clusters (Fig. 7). This reflects the greater variability and diversity of the BES 2 (−1.0 V/NaHCO_3_) and BES 3 (−0.8 V/CO_2_) communities, and a difference between biofilm and planktonic cells was observed, therefore, only in BES 1 (−1.0 V/CO_2_). This indicated that the cathode provided different conditions from catholyte in BES 1 (−1.0 V/CO_2_) leading to enrichment of distinct bacterial communities (Figs. 7 and 8).

The most notable feature of the cathodic biofilm communities in BES 1 (−1.0 V/CO_2_), which showed the highest levels and most diverse range of organic products was enrichment of *Acetobacterium*. *Acetobacterium* also accounted for one of the major differences between the cathodic biofilm community and planktonic cells from the catholyte of BES 1 (*p*-value = 5.7 × 10^−5^). *Acetobacterium* was enriched extensively in the biofilm of BES 1 (46.4 ± 7.01% of reads) compared to the catholyte (7.2 ± 0.9 % of reads) (Fig. 8). The relative abundance of *Acetobacterium* in the biofilm of BES 1 (−1.0 V/CO_2_) was also considerably higher than in BES 2 (−1.0 V/NaHCO_3_) biofilm samples (17.3 ± 10.3 %) and BES 3 (−0.8 V/CO_2_) biofilm samples (8.9 ± 7.0 %) (Fig. 8), consistent with much lower acetate production observed in BES 2 (−1.0 V/NaHCO_3_) and BES 3 (−0.8 V/CO_2_) (Fig. 2). The differences in relative abundance are even more pronounced when it is considered that the cell density on the cathode of BES 1 (−1.0 V/CO_2_) is over an order of magnitude greater than on the cathode of BES 3 (−0.8 V/CO_2_) (Supplementary Fig. 9). Higher abundance of *Acetobacterium* in BES 1 (−1.0 V/CO_2_) than BES 2 (−1.0 V/NaHCO_3_) could be due to either: (1) preferential use of CO_2_ rather than bicarbonate as an inorganic carbon source, or (2) the lower pH in BES 1 (−1.0 V/CO_2_) resulting from addition of gaseous CO_2_ rather than bicarbonate to the medium. Slightly acidic conditions not only provide optimal growth conditions for acetogens, but also could enhance hydrogen production at the cathodes by providing abundant protons^78^. Interestingly, there was no relationship between pH and the relative abundance of *Acetobacterium* (Supplementary Fig. 11; *p*-value ≥ 0.05) suggesting that the greater abundance of *Acetobacterium* in BES 1 (−1.0 V/CO_2_) compared to BES 2 (−1.0 V/NaHCO_3_) was not due to differences in pH in these reactors, but is related to CO_2_ being a more favourable inorganic carbon source than bicarbonate. The importance of the inorganic carbon source has not been explicitly considered in MES processes before, however, gaseous CO_2_ may be a more favourable inorganic carbon source than bicarbonate for some acetogens such as *Clostridium kluyveri* where CO_2_ is known to be required for protein synthesis^68,69^.

Lower enrichment of *Acetobacterium* in biofilms compared to planktonic cells in the catholyte during MES processes has been reported previously^25,49,78^, while more significant enrichment of *Acetobacterium* in biofilms compared to planktonic catholyte communities was reported by Marshall et al.^18^ *Acetobacterium* is a known acetogen able to produce acetate from CO_2_/H_2_, formate and sugars through the Wood–Ljungdahl pathway. Nevin et al. reported lack of direct electron uptake from the cathode by *Acetobacterium woodii* and consequently lack of acetate production by MES^31^. It was reported that *Acetobacterium* is not able to uptake electrons directly from the cathodes as it does not have cytochromes^79^. It is, therefore, considered to be non-electroactive. However, *Acetobacterium* has been reported as a predominant organism in the majority of acetate-producing MES systems enriched from complex environmental inocula^14,18,25,78,80,81^. Moreover, Philips et al. demonstrated promotion of corrosion of Fe(0) by different *Acetobacterium* strains, suggesting that electron uptake from Fe(0) was dependent on the biological (enzymatic) formation of H_2_ which was consumed by the bacteria for acetate production^82^. This is in agreement with the findings of Deutzmann et al., who demonstrated that enzymes such as hydrogenases released by microorganisms were responsible for H_2_ production and consequently electron transfer at the cathode of a BES, and the H_2_ produced was consumed for electro-methanogenesis^33^.

A further notable difference between the composition of biofilm and catholyte planktonic communities of BES 1 (−1.0 V/CO_2_) was the high relative abundance of *Pullulanibacillus (ca*. 23%, *p*-value = 0.016*)* and *Rummeliibacillus (ca*. 9%, *p*-value = 0.019*)* in the catholyte (Fig. 8). Both *Pullulanibacillus* and *Rummeliibacillus* were found previously in bioreactors with low pH (pH ≤ 5.0). *Pullulanibacillus* was detected in a hollow-fibre membrane reactor producing acetate from H_2_ and CO_2_ at a pH of 4.8^83^ and *Rummeliibacillus* was found in the catholyte of BES operated with continuous flow of medium, and was associated with production of isopropanol and butyrate (Supplementary Fig. 10)^25^. Similarly, the occurrence of *Pullulanibacilus* and *Rummeliibacillus* in planktonic catholyte communities of BES 1 (−1.0 V/CO_2_) could be related to acidic pH and butyrate and isopropanol detected only in the BES 1 (−1.0 V/CO_2_) reactors.

### Other organisms in cathodic microbial communities

In addition to *Acetobacterium* which was the most abundant bacterium in the BES exhibiting the highest levels of organic products (BES 1; −1.0 V/CO_2_) a range of other acetogens was detected in the BES. These included *Sporomusa* (*p*-value = 2.6 × 10^−3^) which has been shown to be capable of acetate production from CO_2_ in BES^38,84^. The highest abundance (~ 30% of 16S rRNA gene reads) of *Sporomusa* was found in the biofilm of BES 2 (−1.0 V/NaHCO_3_), while it was represented by less than 10% of the reads from the biofilm of BES 1 (−1.0 V/CO_2_). While there was a clear linear correlation between the relative abundance of *Acetobacterium* and acetate production (Supplementary Fig. 12; *p*-value < 10^−4^), the abundance of *Sporomusa* did not correlate with acetate production (p-value > 0.05). It is apparent that the abundance of *Sporomusa* was affected by pH (Supplementary Fig. 11; *p*-value < 0.01), justifying the highest abundance of *Sporomusa* in BES 2 (−1.0 V/NaHCO_3_).

In addition to acetate production that can be attributed to *Acetobacterium* or *Sporomusa*, production of other compounds could be related to *Clostridium* spp. that were detected in the BES. All of the *Clostridium* spp. identified belonged to *Clostridium sensu stricto*. These were present at low abundance (~2%) in both the cathodic biofilm and catholyte of BES 1 (−1.0 V/CO_2_). The difference between the abundance of *Clostridium* and *Acetobacterium* enriched from mixed inocula could be justified by low H_2_ partial pressure in BES. In a recent study^85^, it was discussed that *Acetobacterium* has higher cells growth at low H_2_ pressures (e.g. at the cathode), while *Clostridium* growth requires higher H_2_ partial pressures (e.g. gas fermentation)^85^. The low levels of *Clostridium* spp, could explain the low concentration of longer chain products such as butyrate and butanol that were detected in BES 1 (−1.0 V/CO_2_). The *Clostridium* spp. detected in this study were most closely related to clostridia previously identified in bio-electrochemical or acetogenic reactors. *Clostridium sensu stricto* (ASV 68), which composed almost 2% of the biofilm microbial communities of BES 1 (−1.0 V/CO_2_), was most closely related to *Clostridium aciditolerans* JW-YJLB3 (Supplementary Fig. 10). This organism produces acetate, ethanol, butyrate and caproate from syngas^86–90^. Other *Clostridium* spp. present at lower abundance in BES 1 (−1.0 V/CO_2_) were related to *Clostridium propionicum* and *Clostridium tertium* (Supplementary Fig. 10). In addition, detection of isovalerate in BES 1 (−1.0 V/CO_2_) could be related to the *Prevotella* found in microbial communities of BES 1 (*ca. 3%*). *Prevotella* is known for production of propionate which can be elongated to isovalerate using methanol or ethanol produced in the reactors^55–57^ (see production section).

Another major difference between the microbial communities in in BES 1 (−1.0 V/CO_2_) and BES 2 (−1.0 V/NaHCO_3_) was the significant enrichment of methanogens (*p*-value = 1.5 × 10^−9^) in BES 2 (−1.0 V/NaHCO_3_). Approximately 20% of the BES 2 (−1.0 V/NaHCO_3_) community comprised *Methanobrevibacter*, whereas methanogens were present at very low abundance in biofilm in BES 1 (−1.0 V/CO_2_) and represented less than 2% of the catholyte community (Fig. 8). This indicated that bicarbonate and/or higher pH in BES 2 (−1.0 V/NaHCO_3_) was important for the enrichment of methanogens. Microbial community results suggest that inorganic carbon source has a significant influence on the composition of microbial communities in MES systems. This could be a useful approach to enriching the desired CO_2_-reducing microorganisms on BES cathodes.

Although almost no biofilm was formed at the electrodes of BES 3 (−0.8 V/CO_2_; Fig. 3c), confirming the effect of potential on cathodic biofilm development, single cells observed in these reactors comprised a diverse range of methanogens and fermenters such as *Rhodobacter* and *Methanobacterium* explaining the inferior production of organic compounds by MES in BES 3.

### Possible mechanisms of biotic hydrogen production

**Fig. 9:**
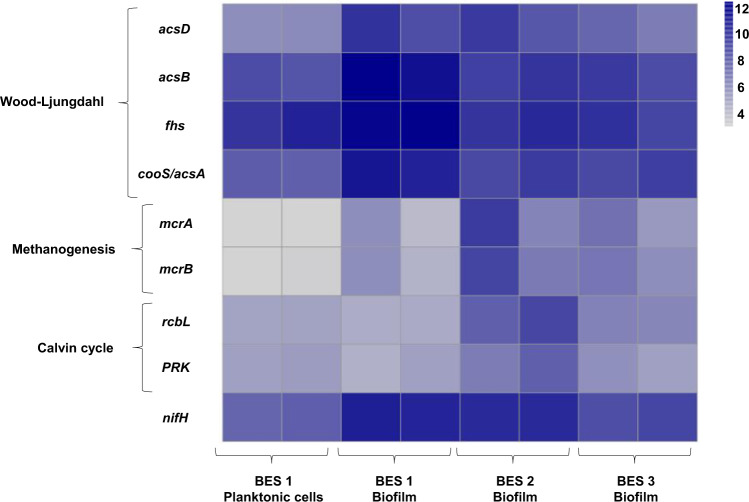
Heatmap of DESeq2 log transformed and normalized counts of *nifH* and key genes of three pathways (Wood–Ljungdahl, Methanogenesis and Calvin cycle) dominating biofilm samples of BES 1 (−1.0 V/CO_2_), BES 2 (−1.0 V/NaHCO_3_), BES 3 (−0.8 V/CO_2_), and planktonic cells of BES 1 (−1.0 V/CO_2_). Darker shade of blue shows the higher relative gene abundance. The key genes are *acsB* (Acetyl-CoA synthase), *acsD* (acetyl-CoA decarbonylase/synthase), *fhs* (fomrate-tetrahydrofolate), *cooS/acsA* (anaerobic carbon-monoxide dehydrogenase catalytic subunit), *mcrA* (Methyl-coenzyme M reductase alpha subunit), *mcrB* (methyl-coenzyme M reductase beta subunit), *rbcl* (ribulose-bisphosphate carboxylase large subunit) and *PRK* (phosphoribulokinase).

Genes encoding *NiFe* or *FeFe* hydrogenases were found in all samples, without any particular pattern. However, higher abundance of *Acetobacerium* in the mature cathodic biofilms from BES 1 (−1.0 V/CO_2_) was associated with abundant *nifH* genes, which were less prevalent in planktonic catholyte communities (Fig. 9). *Acetobacterium* has a nitrogenase (*nif)* gene cluster^80^. *nifH* encodes the subunit of nitrogenase that contains the active site for N_2_ fixation. During N_2_ fixation, one molecule of H_2_ is released for every N_2_ fixed^91,92^. Even in the absence of N_2_, when ammonia is depleted, protons can be reduced to H_2_ by nitrogenase^93^. In the study by Marshall et al.^80^ *nifH* was one of the most expressed genes in an *Acetobacterium* in a BES during MES^80^. The authors suggested that at a sufficiently low cathode potential and low ammonium concentration, production of H_2_ through nitrogenase could provide the reductant and energy source for MES by *Acetobacterium*^80^. Nitrogenase is found in a number of acetogens^94^ and it is possible that nitrogenase in *Acetobacterium* cells is involved in H_2_ production within the cell using *Rnf* complex (membrane-bound Fd:NAD^+^ oxidoreductase). The importance of *Rnf* complex for autotrophic growth of *Acetobacterium* was discussed by Kracke et al.^79^ In *Rnf* complex, NAD^+^ converts to NADH using the electrons from reduced Fd. Due to the difference in redox potentials of Fd and NAD^+^/NADH, extra energy (approximately −20 to −35 kJ mol^−1^) is available for Na^+^-dependent electron transfer in *Acetobacterium*. *Rnf* complex was also reported to be related to nitrogen fixation and the effect of this complex could affect the expression rate of nitrogenase^95^. In addition, electron transfer from the cathode to the *Rnf* complex using Mo–Fe co-factor as a mediator has been studied and reported recently by Milton et al.^96^ Given the growing evidence for H_2_-mediated electron transfer being central in MES, we propose biotic electrons uptake from the cathode could occur through two main operations: 1. Extracellular hydrogen production through *NiFe*/*FeFe* hydrogenases at the cathode surface^80^, oxidation of which is used to generate a proton motive force and energy. 2. Intracellular hydrogen production through the uptake of the electrons from the cathode by a Mo–Fe cofactor that transfers electrons to the *Rnf* complex within the cells. The *Rnf* complex then donates the electrons to nitrogenase for H_2_ production. Alternatively stated, H_2_ production is enhanced by extracellular hydrogenases and in addition electrons may be shuttled, by a Mo–Fe cofactor, to the *Rnf* complex of *Acetobacterium*, that provides reducing power to nitrogenases within the cells, leading to further hydrogen production required for autotrophic growth (Supplementary Fig. 13).

### Functional genes involved in CO_2_ reduction

To clarify the likely pathways driving MES, metagenomic analysis was performed on biofilm and catholyte samples from BES, based on the most abundant (≥10%) members of the BES microbial communities (*Acetobacterium*, *Sporomusa*, *Methanobrevibacter, Rhodobacter*). It is likely that the Wood–Ljungdahl pathway (used by *Acetobacteirum* and *Sporomusa)* should be the most prominent CO_2_-reduction pathway. The presence of *Methanobrevibacter* and *Rhodobacter* indicate that pathways for methanogenic CO_2_-reduction and the Calvin cycle should also be important under some conditions. The abundance of the genes encoding the key enzymes in these pathways was, therefore, quantified in the metagenome datasets (Fig. 9). The proportion of reads from all genes in these three pathways is provided in Supplementary Fig. 14. All the genes involved in the Wood–Ljungdahl pathway (*p*-value = 3.7 × 10^−3^) were present in both cathodic biofilm and catholyte samples (Supplementary Fig. 14). This indicates that the potential for acetogenic CO_2_ reduction existed in the BES operated under all three conditions tested. However, key enzymes of the Wood–Ljungdahl pathway were detected in greater level in the biofilm of BES 1 (−1.0 V/CO_2_). 1.53 ± 0.13% of all the metagenomic reads in biofilm samples of BES 1 (−1.0 V/CO_2_) was assigned as genes involved in the Wood–Ljungdahl pathway. This supports the notion that the Wood–Ljungdahl is a dominant carbon fixation pathway in BES 1 (−1.0 V/CO_2_). Wood–Ljungdahl pathway genes were present at significantly lower abundance in biofilm samples from BES 2 (−1.0 V/NaHCO_3_, 0.8 ± 0.05 %, *p*-value = 0.022), BES 3 (−0.8 V/CO_2_, 0.66 ± 0.15%, *p*-value = 0.012) and in catholyte communities from BES 1 (−1.0 V/CO_2_, 0.53 ± 0.09 %, *p*-value = 0.048) (Fig. 9). This was consistent with the abundance of *Acetobacterium* in the biofilm of BES 1 (−1.0 V/CO_2_) determined from analysis of 16S rRNA genes.

*mcrA* and *mcrB* genes, encoding key enzymes for methanogenesis were present in highest abundance in biofilm samples from BES 2 (Fig. 9). However, they were not detected in catholyte samples from BES 1 (−1.0 V/CO_2_) and were present at low abundance in biofilm samples from BES 1 (−1.0 V/CO_2_). The relative abundance of reads assigned as genes involved in methanogenesis was the highest in biofilm of BES 2 (−1.0 V/NaHCO_3_), (0.71 ± 0.51%) while they were present at lower abundance, 0.22 ± 0.09 % and 0.08 ± 0.02%, in biofilm samples from BES 3 (−0.8 V/CO_2_) and BES 1 (−1.0 V/CO_2_), respectively. Genes for methanogenesis were present in lowest abundance (0.02 ± 0.00%) in catholyte samples from BES 1 (−1.0 V/CO_2_). This indicated lower abundance of methanogens enriched in BES 1 (−1.0 V/CO_2_) which was in agreement with 16S community analysis (Fig. 8).

Genes encoding the key enzymes involved in the Calvin cycle were found in all samples and although these comprised more than 1% of the reads in all samples, this high percentage could be due to the similarity of enzymes such as phosphoglycerate kinase and transketolase in the Calvin cycle with many other pathways of anabolic or catabolic biochemical pathways or biosynthesis metabolisms. In addition, it has to be taken into consideration that analysis was performed using DNA showing only potential function and not the real activity.

To conclude, we showed that applied potential and inorganic carbon source are important parameters in enhancing the efficiency of the system by affecting the development of the biofilm during MES and its microbial composition. The cathodic potential of −1.0 V and gaseous form of CO_2_ provided the most optimal condition for development of the dense biofilm at the cathode dominated by *Acetobacterium*, in which acetate production rate of 11.0 ± 1.6 mM day^−1^ and coulombic efficiency of *ca*. 69% were achieved. Longer chain products of butyrate, butanol and isovalerate (less than 15 mM) were detected only under an applied potential of −1.0 V with CO_2_ as the carbon source. Significant enrichment of *Acetoabcterium* seemed to be related to preferential consumption of CO_2_ in gaseous form rather than the slightly acidic pH provided by supplying CO_2_ in the catholyte. It was shown that the cathode in MES primarily provides electrons which are used to form H_2_ which in turn is used as an energy source by microbial cells for CO_2_ reduction. At a more negative potential, abiotic production of low concentrations of H_2_ was needed to initiate growth of the microbial community and hence biofilm formation at the surface of the electrode, which was further enhanced significantly through enzymatic activity within the biofilm. There was a positive correlation between the density of the biofilm dominated by *Acetobacterium*, the abundance of *nifH*, biotic H_2_ production (from CV) and the production of organic compounds particularly acetate. This proposed the intracellular H_2_ production by nitrogenase in *Acetobacterium* cells using the electrons derived from the cathode, in addition to the known extracellular H_2_ production. The presence of nitrogenase in *Acetobacterium* and its role in autotrophic growth of *Acetobacterium* may explain why *Acetobacterium* and not other acetogens such as *Sporomusa*, is dominantly enriched in bio-electrochemical reactors inoculated with mixed consortia. In addition, the fact that *Acetobacterium* grew principally in the biofilm rather than in suspension was likely related to the enzymatic production of H_2_ needed for MES, at the surface of the cathode. Enzymatic H_2_ production at the cathode enhanced the electron transfer between the cathode and microbial community evidenced by 10-fold decrease in charge transfer resistance of the system. This is the first report in this phenomenon. The CO_2_-reducing biofilm maintained highly active during the long-term experiment even with the stress associated with the low pH due to the accumulation of acidic products.

## Methods

### Bio-electrochemical reactors operation

Reactors used in this study were cubic dual chamber Perspex cells. Each chamber had an internal volume of 80 ml with a 70 ml glass bottle fixed to the top of each chamber as a headspace to allow analysis of headspace gases. A cation exchange membrane (FUMASEP-FKB-PK-130, Fumatech, Germany) was used to separate the anodic and cathodic compartments. Graphite felt (VWR, Cat. No. 43200.RR, Alfa Aesar) with a projected surface area of 4.9 cm^2^ was placed in the cathodic compartment as a cathode, attached to a titanium wire to connect the cathode to the external circuit. A platinum mesh electrode with a similar size to the cathode was connected to titanium wire and used as an anode. A reference electrode with Ag/AgCl saturated in 3 M NaCl (RE-5B, BASi, USA) was used. All the potentials in this study are reported vs. Ag/AgCl.

50% of the cathodic compartment was inoculated with the catholyte of acetate producing half-cells that had been operated in the lab for 100 days with a poised cathode potential of −1.0 V. The initial half-cells had been inoculated with pre-heated activated sludge collected from a wastewater treatment plant (Newcastle upon Tyne, UK). The cathodic medium consisted of K_2_HPO_4_ (0.35 g/L), KH_2_PO_4_ (0.25 g/L), NH_4_Cl (0.25 g/L), KCl (0.5 g/L), CaCl_2_.2H_2_O (0.15 g/L), MgCL_2_.6H_2_O (0.6 g/L), NaCl (1.2 g/L), yeast extract (0.01 g/L), Trace metal solution (1 ml/L), Vitamin solution (2.5 ml/L), Tungstate-selenium solution (0.1 ml/L)^49^. When required sodium 2-bromoethanesulfonate with a final concentration of 5 mM was added to the cathodic medium. When CO_2_ was used as the sole inorganic carbon source, the catholyte was purged with CO_2_/N_2_ (90/10) for 20 min to provide the dissolved inorganic carbon source and remove dissolved oxygen. The final inorganic carbon concentration in the catholyte was 346 ± 20 mg/L measured using a TOC analyser. When HCO_3_^−^ was used as the sole inorganic carbon source, 2.5 g/L NaHCO_3_ was added to the catholyte, providing 357 mg/L inorganic carbon in the solution. To remove the dissolved oxygen in the catholyte prepared with NaHCO_3_, the cathode chamber was purged with N_2_/CO_2_ (90/10) for 20 min before being transferred to the cathodic compartment. For all the reactors, 40% of the catholyte was refreshed every 14–21 days. When the inorganic carbon concentration was less than 30 mg/L, catholytes were purged with CO_2_/N_2_ or NaHCO_3_ was added to the catholyte. After purging both solutions (with CO_2_ or NaHCO_3_), the pH was 6.9 ± 0.1. The anodic chamber was filled with the same medium used as the cathodic medium except for the absence of inoculum, trace mineral, vitamin and Tungstate-selenium solutions. Anolyte was continuously purged with N_2_ to remove the oxygen produced throughout the experiment in this compartment.

BESs were operated in duplicate with a 3 electrode configuration. The cathode potential was controlled using a potentiostat (Quad Potentiostat, Whistonbrook Technologies, UK) and the cathodic current was monitored. To compare the effect of cathodic potential on MES processes, two different potentials of −0.8 and −1.0 V were applied, using CO_2_ as the sole inorganic carbon source. The potentials were selected based on a preliminary experiment conducted for 2 weeks under sterile condition where abiotic H_2_ production was observed only at −1.0 V. Additionally, to understand the effect of inorganic carbon source on MES, BES operated with CO_2_ or NaHCO_3_ were compared, using a cathodic poised potential of −1.0 V. Therefore, three different conditions were tested: BES1 (−1.0 V/CO_2_), BES2 (−1.0 V/NaHCO_3_) and BES3 (−0.8 V/CO_2_). All BES were started at the same time and operated in parallel at a temperature of 35 °C.

To confirm the effect of the cathodic applied potential as an energy source on the bacterial growth and metabolism, two inoculated reactors were operated under open circuit potential (OCP). Production of organic compounds by MES and biofilm development in BES 1, BES 2, and BES 3 and control experiments were compared.

### Chemical analyses

5 ml liquid samples were collected and filtered (pore size: 0.2 µm) from the catholyte of BESs every 3–5 days including control reactors. Concentrations of carboxylic acids (formate, acetate, propionate, iso-butyrate, butyrate, iso-valerate, valerate and hexanoate) in the catholytes at the time of sampling were measured using ion chromatography (Eco IC, Metrohm, Switzerland) equipped with a METROHM 6.1005.200 column and autosampler. Concentrations of acetone and alcohols (methanol, ethanol, isopropanol, butanol and hexanol) were measured using gas chromatography (GC-2010, Shimadzu Tracera, Japan) equipped with a Barrier Discharge Ionization (BID) detector and autosampler. A Zebron ZB-WAXplus capillary column (Phenomenex) was used for separation of acetone and alcohols. To detect the gas production in the reactors (H_2_, N_2_, O_2_, CH_4_ and CO_2_), 0.5 ml gas sample collected from the cathode headspace was injected into a gas chromatograph (GC-2010, Shimadzu Tracera, Japan) immediately after collection from the reactors. The GC was equipped with a Barrier Discharge Ionization detector (BID) and an ASI-5000 autosampler. The column used for the gas separation was a ShinCarbon ST micropacked column 80/100 (Restek) using Helium as a carrier gas at a pressure of 100 kPa. A TOC 5050 A Total Organic Carbon analyser (Shimadzu, Japan) was used to measure total inorganic carbon (TOC) in the catholyte. 4 ml of 1/5 diluted samples were loaded into vials in the ASI-5000 autosampler. The concentration of total carbon (TC) as CO_2_, following combustion at 800 °C, was measured with an infrared detector, and total inorganic carbon (TIC) was removed and measured by acidifying the samples. TOC was measured periodically throughout the experiment to ensure that there were no additional organic compounds produced that were not detected in the analysis of carboxylic acids and alcohols. TOC was calculated by difference as follows: TOC = TC – TIC. pH of catholyte samples was measured, using a HI 9025 pH meter (Hanna Instruments, USA) with a pH probe (11542543; Fisher-Scientific, UK) calibrated between 2.0 and 9.0.

### Electrochemical analyses

To gain better understanding about the electrochemical activity of the biocathodes and electron transfer between the cathode and bacteria, cyclic voltammetry (CV) was performed at the end of the experiment using an Autolab PGSTAT203 potentiostat and NOVA electrochemistry software (Metrohm, Switzerland). A scan rate of 2 mV s^−1^ was selected with potential steps of 0.5 mV. To perform CVs, 50% of the catholyte was replaced with fresh medium, and the pH was adjusted to 6.7 ± 0.1. Voltammograms of the second scan were compared to scans of the plain electrode performed before biofilm development.

Electrochemical impedance spectroscopy (EIS) was applied to BES 1 after 104 days and to the plain electrode to determine the effect of biofilm on ohmic and charge transfer resistances. EIS was performed using a 3 electrode configuration and applying a potential of −1.0 V to the cathode. Frequencies from 10,000 to 0.1 Hz were applied with an amplitude of 10 mV.

### Cathodic biofilm imaging

At the end of the experiment, 2 pieces (1 cm × 1 cm) of electrode were sampled from each BES using a sterile razor blade. A confocal microscope (Zeiss AxioObserver LSM800/SDI) was used to determine the coverage, distribution and volume of dead and live cells in the cathodic biofilm using Zeiss ZEN imaging software (Blue Edition, version 2.5). To ensure accurate measurement of live cells, samples were prepared and stained directly after terminating the BES experiments. Samples were stained using the dead-live staining kit (FilmTracer LIVE/DEAD Biofilm Viability Kit, Invitrogen). Confocal images were collected on three different channels: SYTO 9 for live cells, propidium iodide for dead cells and reflectance for visualization of the graphite fibres. Three different fields of view were selected randomly for each sample and all the quantitative analysis are reported as an average from the three fields of view. A z-scan was conducted from 70 to 200 µm of depth with a step size of 5 µm. The percentage of live and dead cells was extracted from the stacks of z-scan images and measured using the Huygens software (Scientific Volume Imaging, The Netherlands). To remove background noise, an intensity threshold was set for all the channels using the Huygens software. The threshold value for each channel was determined from control samples of stained, sterile electrodes using the software supplied with the microscope (ZEN blue, version 2.5). The dead and live bio-volume was calculated with respect to the total image size in each field of view.

Scanning electron microscopy (SEM) of the cathodic biofilm was also conducted. A Tescan Vega 3LMU SEM fitted with a Bruker XFlash 6 | 30 detector for energy-dispersive X-ray spectroscopy (EDS) analysis was used. Two 1 cm × 1 cm samples of cathode were prepared for SEM by fixing in 2% Glutaraldehyde for 24 h, rinsing in the original medium matrix and dehydrating in a graded series of ethanol. Electrodes were dried using critical point drying and finally coated with gold in a SEM coating unit.

### Cell counts

At the end of the experiment, 2 pieces (1 cm × 1 cm) of electrode and 2 mL of catholyte were sampled from each BES. Flow cytometry (Accuri C6 flow cytometer, BD Biosciences) was used to count the number of the cells including both dead and live cells in the cathodic solutions and cell suspensions prepared from the electrode samples. Samples for cell counts were fixed in 2 mL of 50% ethanol after collection. In order to count the number of cells in biofilms, electrode samples were crushed using a sterile stainless steel rod. Crushing the electrodes allowed the biofilm to detach bacteria of the electrode, giving a homogenous sample of suspended bacterial cells. Samples were then prepared for flow cytometry following sample preparation procedures described by Vignola et al.^97^. and stained by SYTO 9 green fluorescent nucleic acid stain. Electronic gating was performed using the negative control (stained sterile filtered EDTA) to separate the signals from the cells from background noise.

### Microbial community analysis

At the end of the experiment, microbial community analysis was performed on the catholyte and cathode samples to determine the composition of the cathodic biofilm communities. DNeasy PowerWater Kit (14900-100-NF, QIAGEN, Germany) was used to extract DNA from the samples. 2 mL of catholyte were sampled in duplicate and filtered using a 0.2 µm pore-size nylon membrane filter (Swinnex, China). The filters were transferred to lysis tubes from the DNA extraction kit. Duplicate electrode samples (1 cm×1 cm) were directly transferred to lysis tubes. The V4 region of the 16 S rRNA gene was sequenced using an Illumina MiSeq sequencer using V2 chemistry (pair-end read length: 2 × 250 bp), to determine the composition of the microbial communities. DNA sequencing was carried out by the NU-OMICs Facility, Northumbria University, UK. 10659 to 65345 raw reads per sample were obtained. The data from the Illumina MiSeq were processed using the ‘’Dada 2” package^98^ in R^99^. 4.2 ± 1.2% of the sequences were discarding during quality filtering. The sequences of each amplicon sequence variant (ASV) were assigned to a taxonomic group using the Ribosomal Database Project (RDP) reference database^100^ with a bootstrap confidence level of 80 (97.6% of sequences assigned at the genus level). After taxonomic assignment, the relative abundance of each taxon was determined from kingdom to genus levels using the ‘’phyloseq” package^101^. To compare differences between the samples, Bray–Curtis distances were calculated between samples. Non-metric multidimensional scaling (NMDS) and hierarchical clustering was conducted from the Bray–Curtis dissimilarity matrix using the package “vegan” in R^102^. MANOVA and ANOVA tests were performed using the package “stats” in R^103^. Following that, selected sequences were incorporated in phylogenetic trees using the ‘’ape” package after sequence alignment using the ‘’msa” package in R^104–106^. To evaluate the accuracy of the community analysis, a mock bacterial community with a defined composition (ZymoBIOMICS® Microbial Community Standard) was used as a positive control.

### Shotgun metagenome sequencing

Metagenomes were sequenced using an Illumina MiSeq sequencer in order to detect gene encoding pathways potentially involved in extracellular electron transfer and organic product synthesis. DNA sequencing was carried out by the NU-OMICs Facility, Northumbria University, UK. DNA samples extracted from cathode biofilms and catholyte samples was diluted to 0.2 ng/μL with molecular biology grade water. Biofilm from 6 electrodes of each duplicate BES and 2 catholyte samples from duplicates BES 1 were analysed. Only catholyte samples from BES 1 were used for shotgun metagenome sequencing, as 16 S rRNA analysis showed difference in the bacterial communities enriched in the biofilm and planktonic cells from these reactors while in BES 2 the communities were similar. Detailed procedure of the shotgun metagenome sequencing can be found in the Supplementary information.

Raw FASTQ files from the MiSeq were uploaded to MG-RAST for analysis using the default pipeline^107^. Count tables of KEGG Orthology (KO) identifiers were downloaded from MG-RAST and amalgamated for all eight samples. Gene counts derived from MG-RAST were imported into DESeq2^108^ in R^99^. The DESeq2 pipeline was performed on the count data with the Wald test and parametric fit type. The DESeq2 function “VarianceStabilizingTransformation” was performed on the DESeq2 data, to generate log-transformed and library size-normalized gene counts. Due to a lack of triplicate samples within reactor groups, differential abundance of KOs was not performed. Instead, key target KOs were extracted from the DESeq2 normalised and transformed count table and their abundances investigated. The presence of genes involved in the metabolic pathways related to extracellular electron transfer and organic product synthesis were interrogated using the KEGG pathway mapping tool in MG-RAST. In particular, three pathways were investigated: Calvin cycle (M00165), Methanogenesis (M00356) and Wood–Ljungdahl (M00377). These pathways were selected according to the dominant members of the bacterial community enriched in BES revealed through 16 S rRNA analysis. Acetyl-CoA synthase (*acsB*, K14138), acetyl-CoA decarbonylase/synthase (*acsD*, K00194), formate-tetrahydrofolate (*fhs*, K01938) and anaerobic carbon-monoxide dehydrogenase catalytic subunit (*cooS/acsA*, K00198) were selected as the key enzymes that are involved in carbon fixation through the Wood–Ljungdahl pathway. Methyl-coenzyme M reductase alpha subunit (*mcrA*, K00399) and methyl-coenzyme M reductase beta subunit (*mcrB*, K00401) are key enzymes catalysing methane production and were selected as representative of genes for Methanogenesis, and ribulose-bisphosphate carboxylase large subunit (*rbcl*, K01601) and phosphoribulokinase (*PRK*, K00855) were selected for carbon fixation through the reductive pentose phosphate or Calvin cycle as the first enzymes involved in the pathway. The R package “pheatmap”^109^ was used to generate heatmaps of the DESeq2 log-transformed and normalized counts of target genes. Additionally, raw FASTQ files were analysed using GraftM^110^ with gene packages that targeted *NiFe* hydrogenase and *FeFe* hydrogenase.

### Reporting summary

Further information on research design is available in the Nature Research Reporting Summary linked to this article.

## Supplementary information

Supplementary Information

Reporting Summary

## Data Availability

All the sequencing reads from metagenomic and 16S rRNA analyses have been deposited in the NCBI SRA database under Bioproject PRJNA663785. Additional metadata are available at 10.25405/data.ncl.12993812.v2.
