# Supplementary material for: Parameters influencing the development of highly conductive and efficient biofilm during microbial electrosynthesis: the importance of applied potential and inorganic carbon source
Source: NPJ Biofilms Microbiomes. 2020 Oct 14;6:40. doi: 10.1038/s41522-020-00151-x (PMC7560852; doi:10.1038/s41522-020-00151-x)
Supplement: Supplementary file 1 — Supplementary Information [file 41522_2020_151_MOESM1_ESM.pdf]

Supplementary information

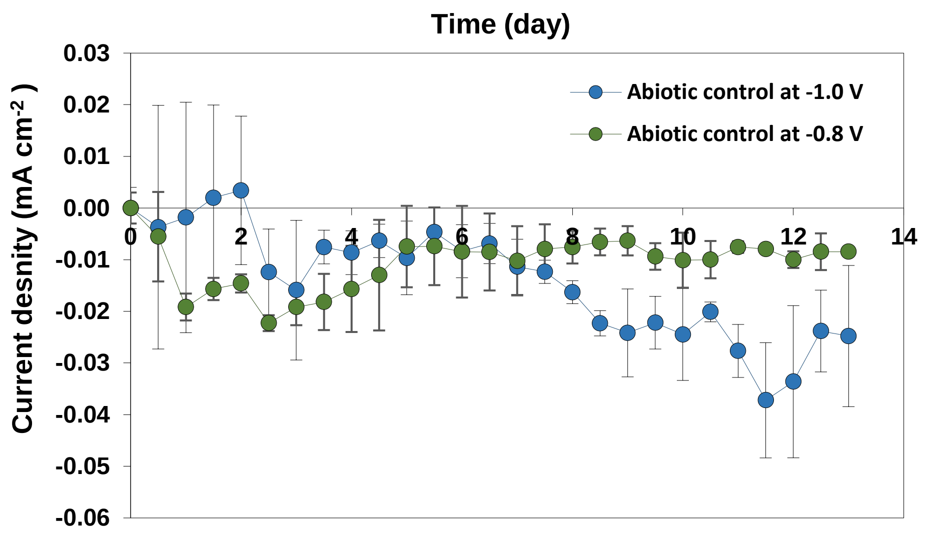

Supplementary Fig. 1. Cathodic current consumption of abiotic control reactors with the applied potentials of -1.0 V and -0.8 V. The error bars represent the standard deviation calculated from duplicate samples in two reactors.

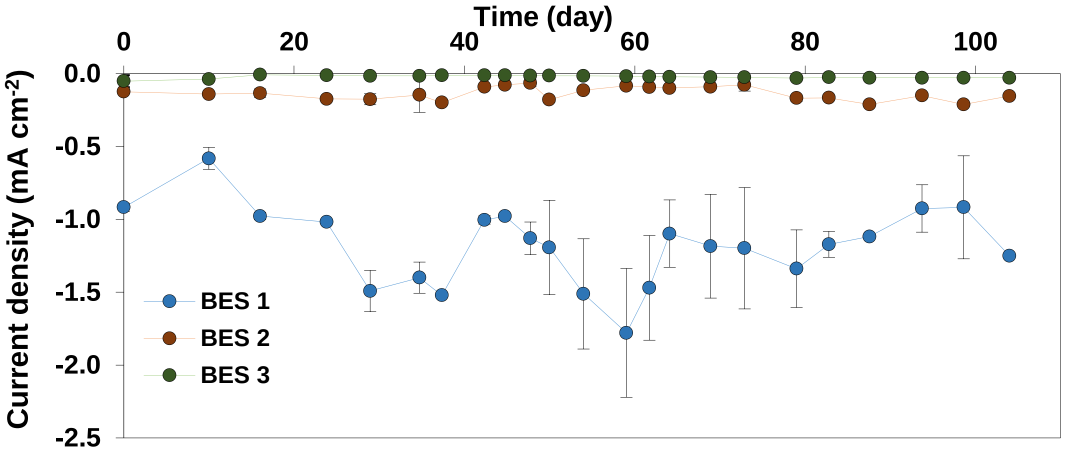

Supplementary Fig. 2. Cathodic current consumption of BESs 1 (-1.0 V/CO_2), BESs 2 (-1.0 V/$NaHCO_3$) and BESs 3 (-0.8 V/CO_2) during the experiment. The error bars represent the standard deviation calculated from duplicate samples in two reactors.

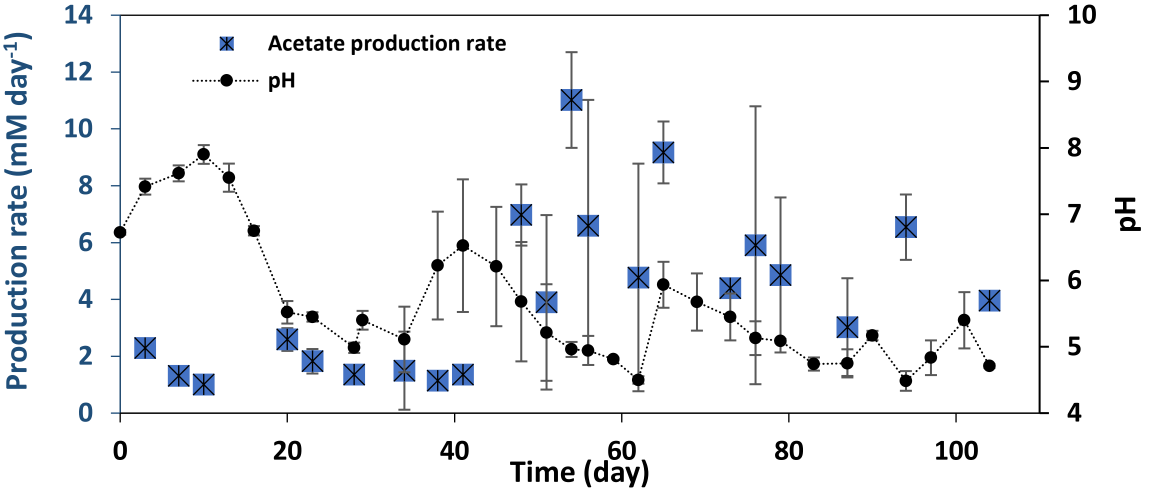

Supplementary Fig. 3. Acetate production rate in BES 1 (-1.0 V/CO_2) during 104 days of the experiment. The error bars represent the standard deviation calculated from duplicate samples in two reactors.

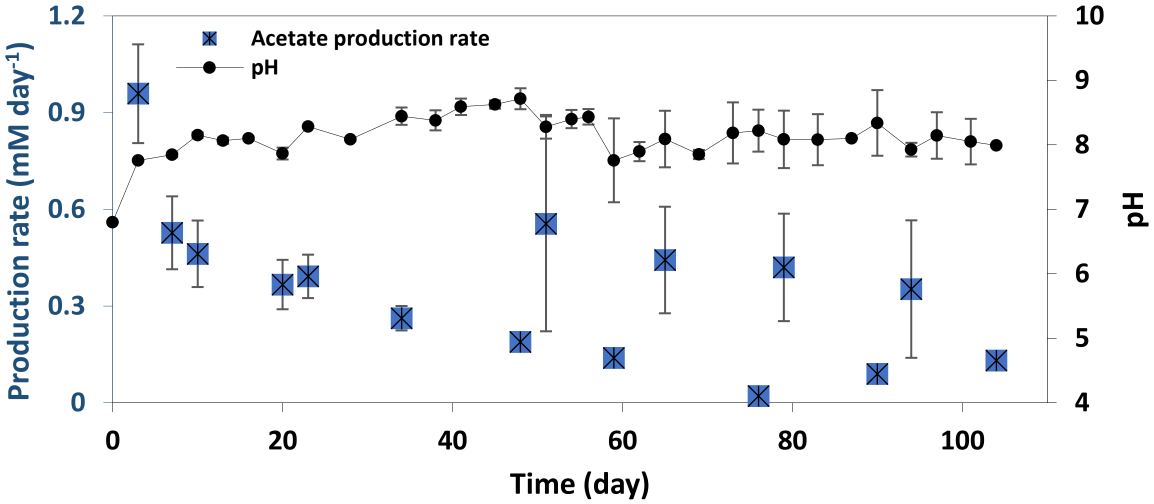

Supplementary Fig. 4. Acetate production rate in BES 2 (-1.0 V/$NaHCO_3$) during 104 days of the experiment. The error bars represent the standard deviation calculated from duplicate samples in two reactors.

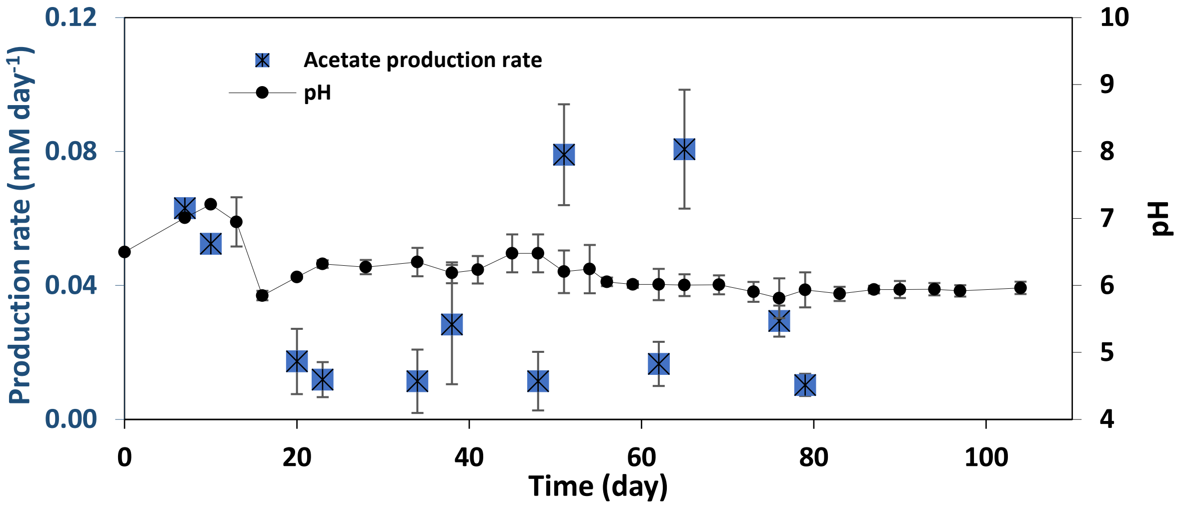

Supplementary Fig. 5. Acetate production rate during the experiment in BES 3 (-0.8 V/CO_2) during 104 days of the experiment. The error bars represent the standard deviation calculated from duplicate samples in two reactors.

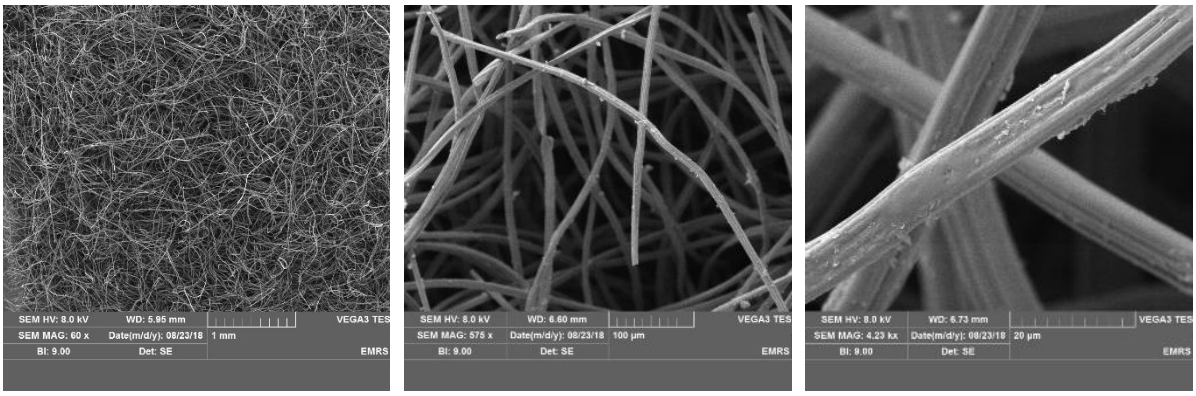

Supplementary Fig. 6. SEM images cathodes following 104 days of operation in BES under open circuit potential condition. From left to right the scale bars represent 1 mm, 100 microns and 20 microns respectively.

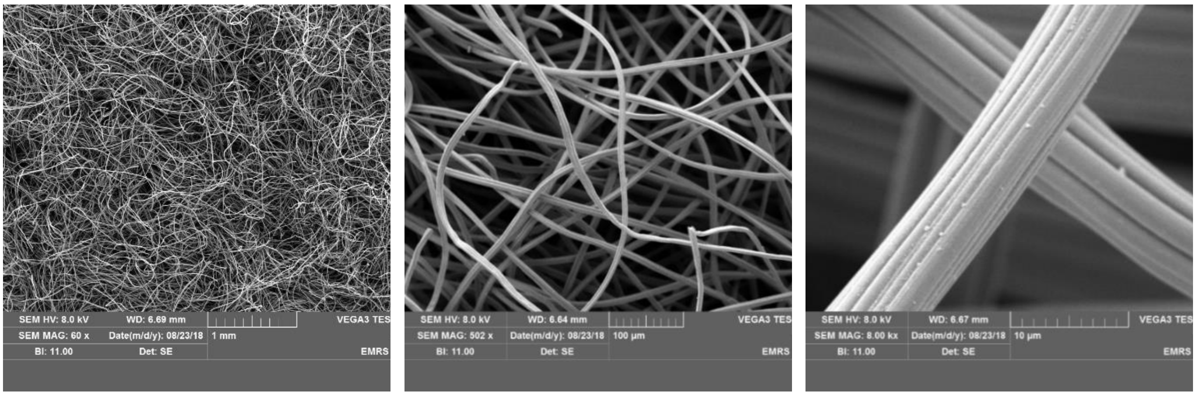

Supplementary Fig. 7. SEM images of the plain graphite felt. From left to right the scale bars represent 1 mm, 100 microns and 10 microns respectively.

(a)

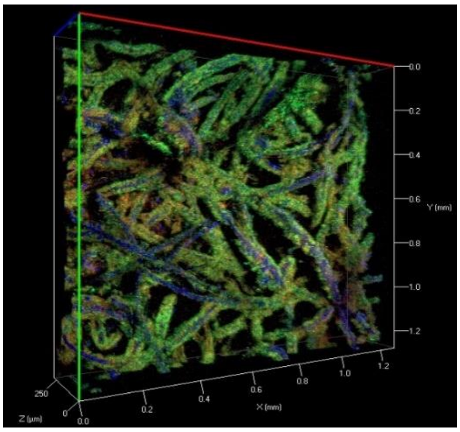

(b)

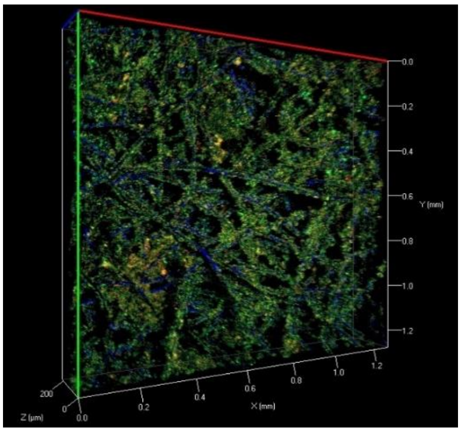

(c)

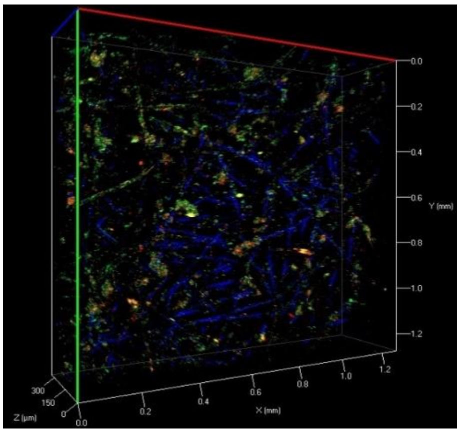

(d)

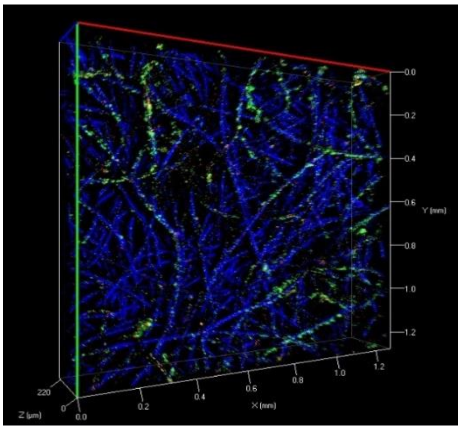

(e)

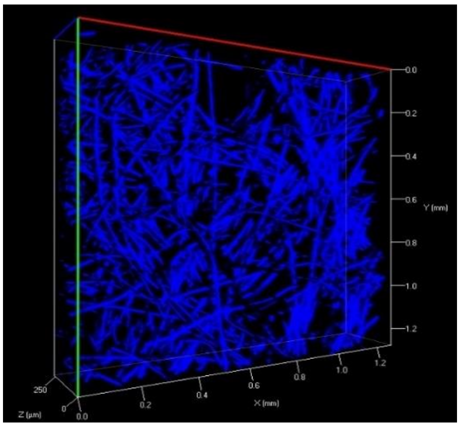

Supplementary Fig. 8. 3D images of the confocal microscopy from the electrodes surface after 104 days of experiment in (a) BES 1 (-1.0 V/CO_2), (b) BES 2 (-1.0 V/$NaHCO_3$), (c) BES 3 (-0.8 V/CO_2), (d) BES operated in OCP, and (e) plain electrode

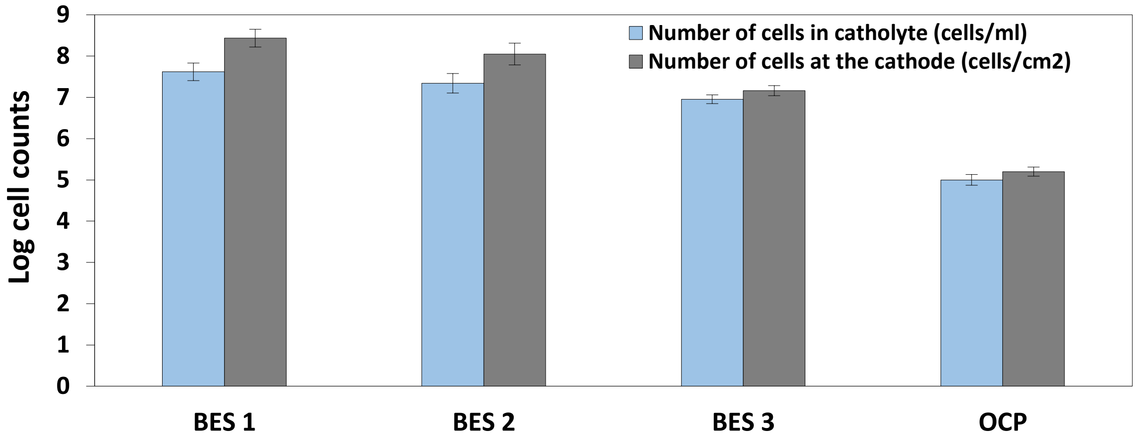

Supplementary Fig. 9. Logarithmic scale of the number of cells in 1 ml of catholytes and at 1 cm^2 projected surface area of the cathodes in BES 1 (-1.0 V/CO_2), BES 2 (-1.0 V/$NaHCO_3$), BES 3 (-0.8 V/CO_2) and biotic control experiment under OCP condition. The number of cells was measured using flow cytometry at the end of the experiment. The error bars represent the standard deviation calculated from duplicate samples in two reactors.

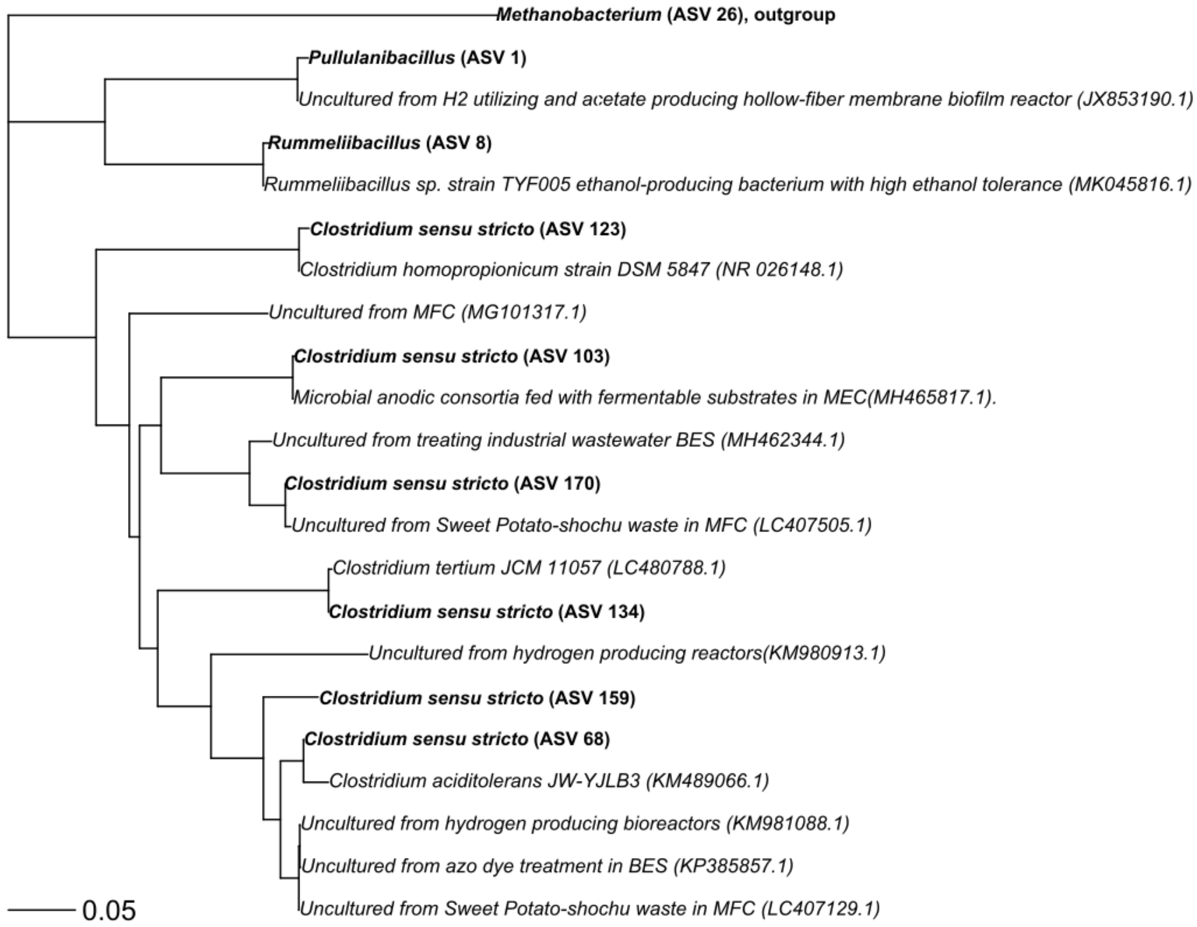

Supplementary Fig. 10. Phylogenetic tree of 16S rRNA gene sequences extracted from community analysis of biofilm and planktonic cells samples from BESs 1 (-1.0 V/CO_2) presented in bold (coded by their ASV numbers) compared to highly similar sequences recovered from NCBI Nucleotide database

collection (coded by their genbank accession numbers). The scale bar shows the number of nucleotide position changes. *Methanobacterium* was used as an outgroup from Archaea.

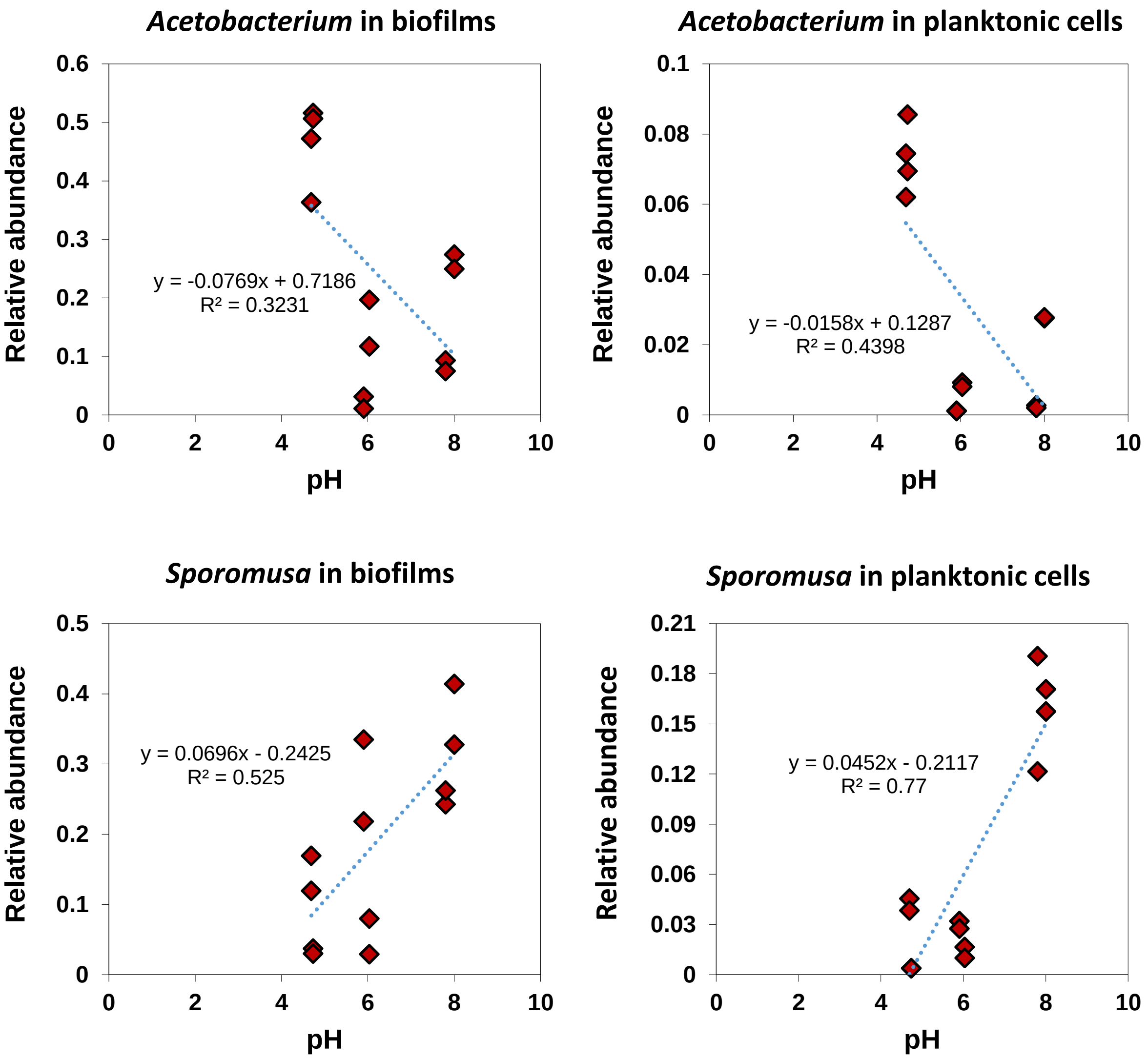

Supplementary Fig. 11. Correlation between the abundance of *Acetobacterium* in biofilm (p-value: 0.0538), *Acetobacterium* in planktonic cells (p-value: 0.0187), *Sporomusa* in biofilm (p-value: 0.0076), *Sporomusa* in planktonic cells (p-value: 1.7×10^{-4}) and pH.

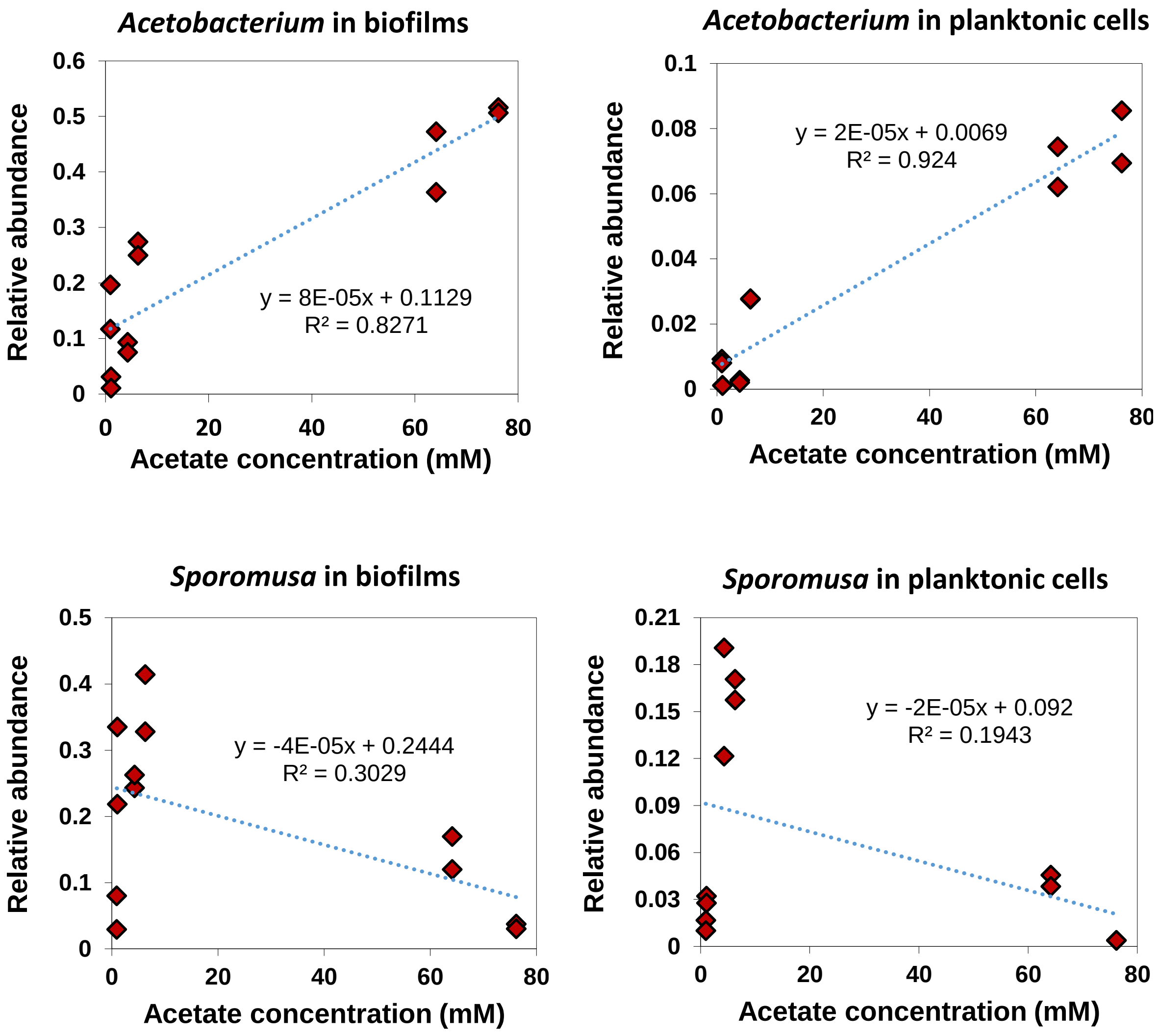

Supplementary Fig. 12. Correlation between the abundance of *Acetobacterium* in biofilm (p-value: 4.1 x 10^{-5}), *Acetobacterium* in planktonic cells (p-value: 6.4 x 10^{-7}), *Sporomusa* in biofilm (p-value: 0.0636), *Sporomusa* in planktonic cells (p-value: 0.1514) and acetate concentration.

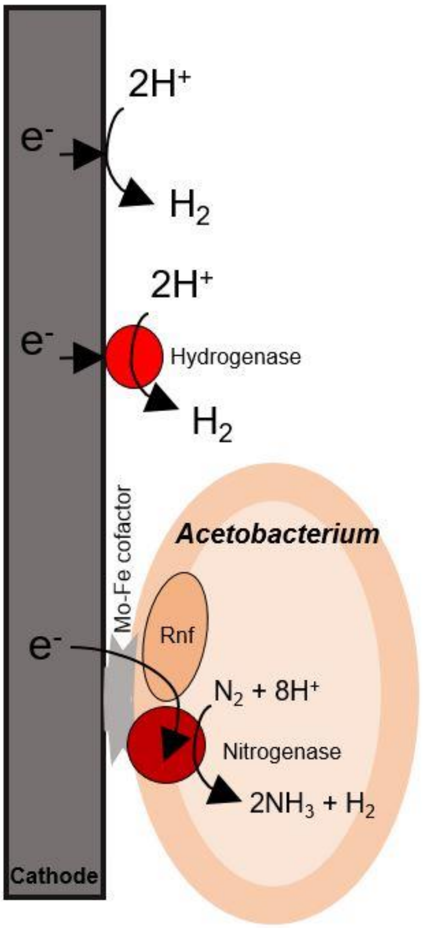

Supplementary Fig. 13. Schematic of abiotic and biotic electrons uptake from the cathode for MES proposed in our study.

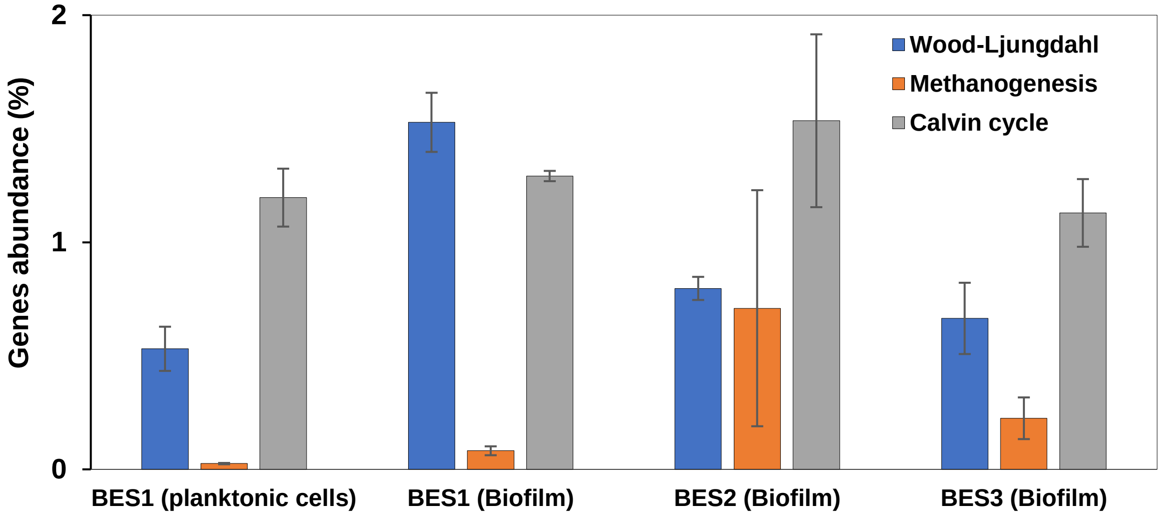

Supplementary Fig. 14. Abundance of all the genes involved in three pathways of Wood-Ljungdahl, Methanogenesis and Calvin cycle presented in the biofilm samples of BESs 1 (-1.0 V/CO_2), BESs 2 (-1.0 V/$NaHCO_3$) and BESs 3 (-0.8 V/CO_2) and planktonic cells of BES 1 (-1.0 V/CO_2).

Calculation of carbon conversion efficiency

Carbon balance was performed considering the consumption of CO_2 or HCO_3^- and generation of the products over each batch cycle, from the beginning of the cycle until refreshing the medium, according to equation (1).

$$\text{Carbon recovery } (\%) = \left(\frac{(P2-P1)}{C2-C1} \times n\right) \times\ 100 \qquad (1)$$

Where C1 was the concentration (M) of CO_2 or HCO_3^- at the beginning of the cycle, C2 was the concentration (M) of CO_2 or HCO_3^- at the end of the cycle, P1 was the concentration (M) of the product remained from the previous batch cycle, P2 was the concentration (M) of the product at the end of the cycle, and n was the ratio of CO_2 or HCO_3^- to the product according to the stoichiometry of the reaction. In case of acetate production, the major product detected in this study during MES, 2 mol of CO_2 is required to produce one mol acetate (equation 2). Therefore, n is equal to 2.

$$2\ HCO_3^- + 9\ H^+ + 8e^- \rightarrow CH_3COO^- + 4\ H_2O \qquad (2)$$

In the last batch cycle of BES 2 (-1.0 V/$NaHCO_3$), for instance, 8 mM HCO_3^- were consumed in both replicates, while 1.87 and 2.40 mM acetate was produced in the replicate 1 and replicate 2, respectively. Therefore, the carbon recovery were calculated 46.6% and 60.0% for replicate 1 and replicate 2 respectively according to equation (1), or 53.3 ± 9.4% in average of the replicates for the last batch cycle. To calculate the total carbon conversion efficiency during 104 days of experiment, the average and standard deviation of the carbon recovery for the duplicates in all 8 batch cycles were calculated (n = 16).

Detailed procedure of shotgun metagenome sequencing

Illumina MiSeq sequencer was used for metagenomes sequencing. Ligation and tagging of reads ('tagmentation', which includes fragmentation of genomic DNA) was performed on metagenomic DNA using a NextEra XT DNA Library Preparation Kit (Illumina, USA) following the manufacturer's protocol, which also adds Illumina-specific adaptor sequences to the ends of DNA fragments. Tagmented DNA was amplified *via* a limited-cycle PCR to add Illumina-specific index sequences – index 1 (i7) and index 2 (i5) – which enables cluster formation. Indexed and tagmented DNA was purified using AMPure XP magnetic beads (Agencourt, UK). Resuspension buffer was added to the PCR amplified DNA and mixed to resuspend the magnetic beads. 13 µl of supernatant containing eluted DNA was transferred. DNA amplicon fragments were then checked for fragment size using an Agilent 2100 Bioanalyzer (Agilent Technologies, USA) using an Agilent High Sensitivity DNA chip. DNA was quantified using a Qubit 3.0 fluorometer. Indexed DNA fragments were diluted to an equimolar library mix of sample DNA with a pooled concentration of 4 nM. The sample library was denatured with 0.2 N NaOH, incubated at room temperature for 5 minutes, and HT1 buffer added. A final 20 pM denatured library was then diluted to 14 pM with additional HT1 buffer and placed on a MiSeq V3 600 bp reagent cartridge for sequencing.
