# Supplementary material for: Parameters influencing the development of highly conductive and efficient biofilm during microbial electrosynthesis: the importance of applied potential and inorganic carbon source
Source: NPJ Biofilms Microbiomes. 2020 Oct 14;6:40. doi: 10.1038/s41522-020-00151-x (PMC7560852; doi:10.1038/s41522-020-00151-x)
Supplement: Supplementary file 2 — Reporting Summary [file 41522_2020_151_MOESM2_ESM.pdf]

nature research

Corresponding author(s): DBPR NPJBIOFILMS-00780R

Last updated by author(s): Sep 17, 2020

Reporting Summary

Nature Research wishes to improve the reproducibility of the work that we publish. This form provides structure for consistency and transparency in reporting. For further information on Nature Research policies, see our Editorial Policies and the Editorial Policy Checklist.

Statistics

For all statistical analyses, confirm that the following items are present in the figure legend, table legend, main text, or Methods section.

n/a	Confirmed	
☐	☒	The exact sample size (*n*) for each experimental group/condition, given as a discrete number and unit of measurement
☐	☒	A statement on whether measurements were taken from distinct samples or whether the same sample was measured repeatedly
☐	☒	The statistical test(s) used AND whether they are one- or two-sided<br>*Only common tests should be described solely by name; describe more complex techniques in the Methods section.*
☐	☒	A description of all covariates tested
☐	☒	A description of any assumptions or corrections, such as tests of normality and adjustment for multiple comparisons
☐	☒	A full description of the statistical parameters including central tendency (e.g. means) or other basic estimates (e.g. regression coefficient) AND variation (e.g. standard deviation) or associated estimates of uncertainty (e.g. confidence intervals)
☐	☒	For null hypothesis testing, the test statistic (e.g. *F*, *t*, *r*) with confidence intervals, effect sizes, degrees of freedom and *P* value noted<br>*Give P values as exact values whenever suitable.*
☐	☒	For Bayesian analysis, information on the choice of priors and Markov chain Monte Carlo settings
☐	☒	For hierarchical and complex designs, identification of the appropriate level for tests and full reporting of outcomes
☐	☒	Estimates of effect sizes (e.g. Cohen's *d*, Pearson's *r*), indicating how they were calculated

Our web collection on statistics for biologists contains articles on many of the points above.

Software and code

Policy information about availability of computer code

Data collection	The data for this study was collected using the software supplied to each specific analysis. The electrochemical analysis using Autolab PGSTAT203 potentiostat were collected by NOVA electrochemistry software (Metrohm,Switzerland). Data from Confocal microscopy were collected by the software of ZEN blue, version 2.5).
Data analysis	The data were analysed using the software that collected the raw data. For confocal microscopy additional software of Huygens (Scientific Volume Imaging, The Netherlands) was also used. The rest of the data were analysed using R package.

For manuscripts utilizing custom algorithms or software that are central to the research but not yet described in published literature, software must be made available to editors and reviewers. We strongly encourage code deposition in a community repository (e.g. GitHub). See the Nature Research guidelines for submitting code & software for further information.

Data

Policy information about availability of data

All manuscripts must include a data availability statement. This statement should provide the following information, where applicable:

- Accession codes, unique identifiers, or web links for publicly available datasets
- A list of figures that have associated raw data
- A description of any restrictions on data availability

All the sequencing reads from metagenomic and 16S rRNA analyses have been deposited in the NCBI SRA database under Bioproject PRJNA663785.

Field-specific reporting

Please select the one below that is the best fit for your research. If you are not sure, read the appropriate sections before making your selection.

☐ Life sciences ☐ Behavioural & social sciences ☒ Ecological, evolutionary & environmental sciences

For a reference copy of the document with all sections, see nature.com/documents/nr-reporting-summary-flat.pdf

Ecological, evolutionary & environmental sciences study design

All studies must disclose on these points even when the disclosure is negative.

Study description	Studying the effect of inorganic carbon source and cathodic potentials on the biofilm development in microbial electrosynthesis in bio-electrochemical system
Research sample	When required, samples were collected from the planktonic cells and biofilms in bio-electrochemical reactors
Sampling strategy	Reactors were in replicate. Samples were collected from each reactor and the average of the analysis were reported with standard deviation. In microbial analysis, at least two samples were collected from each reactor.
Data collection	The data were monitored through the softwares supported each analysis, and were collected by the first author.
Timing and spatial scale	In order to show the production in the reactors, samples for products analyses were collected every 3-5 days, considering enough time for the microbial production. The samples from the biofilm were collected after terminating the reactors at the end of experiment, as they were required throughout the experiment.
Data exclusions	No data were excluded from the analyses.
Reproducibility	The new setup of the reactors were started using the culture in this study and similar results were observed in bio-production and microbial analysis, which the results are presented in a different research report.
Randomization	No randomization was required in our study.
Blinding	No data blinding was required in our study.

Did the study involve field work? ☐ Yes ☒ No

Reporting for specific materials, systems and methods

We require information from authors about some types of materials, experimental systems and methods used in many studies. Here, indicate whether each material, system or method listed is relevant to your study. If you are not sure if a list item applies to your research, read the appropriate section before selecting a response.

Materials & experimental systems

n/a	Involved in the study	
☒	☐	Antibodies
☒	☐	Eukaryotic cell lines
☒	☐	Palaeontology and archaeology
☒	☐	Animals and other organisms
☒	☐	Human research participants
☒	☐	Clinical data
☒	☐	Dual use research of concern

Methods

n/a	Involved in the study	
☒	☐	ChIP-seq
☐	☒	Flow cytometry
☒	☐	MRI-based neuroimaging

Flow Cytometry

Plots

Confirm that:

☐ The axis labels state the marker and fluorochrome used (e.g. CD4-FITC).

☐ The axis scales are clearly visible. Include numbers along axes only for bottom left plot of group (a 'group' is an analysis of identical markers).

☐ All plots are contour plots with outliers or pseudocolor plots.

☒ A numerical value for number of cells or percentage (with statistics) is provided.

Methodology

Sample preparation	Flow cytometry was used in this study to only count the number of the microbial cells in the planktonic cells and biofilms. In order to count the number of the cells at the electrode after disassembling the cells, the electrodes were crushed using the sterile stainless steel rod. Crushing the biocathode allowed the biofilm to remove from the spongy structure of graphite felt, giving the homogenous liquid sample. Samples were prepared for flow cytometry by adding 100 µL surfactant (5% Tween 80 and 10 mM sodium pyrophosphate) to 900 µL sample and shaking it gently, diluting the samples ×200 in filtered sterile Ethylenediaminetetraacetic acid (EDTA) solution, staining the samples by 10 µL SYTO 9 (FilmTracer LIVE/DEAD Biofilm Viability Kit, Invitrogen) solution (diluted ×50 in Dimethyl sulfoxide) and incubating the stained samples in the dark at 60 °C for an hour.
Instrument	Flow cytometry with Accuri C6 flow cytometer (BD Biosciences) was used to count the number of the cells in the cathodic solutions and electrodes.
Software	The software supplied by Accuri C6 flow cytometer (BD Biosciences) was used for collecting the data.
Cell population abundance	Purity of the samples were checked through the gating strategy.
Gating strategy	Desired gates and regions were typically placed through forward scatter (FSC). FSC represented the size of the bacterial cells, providing the proper method for detection of particles. Gating was performed through FSC using stained filtered sterile water to ensure removing the background noise caused by machine or stain.

☐ Tick this box to confirm that a figure exemplifying the gating strategy is provided in the Supplementary Information.
